# A supervised machine learning approach with feature selection for sex-specific biomarker prediction

**DOI:** 10.1038/s41540-025-00523-z

**Published:** 2025-07-01

**Authors:** Luke Meyer, Danielle Mulder, Joshua Wallace

**Affiliations:** 7 Long Tom Place Kanonberg Bellville Western Cape, Siriuz Pty Ltd., Cape Town, South Africa

**Keywords:** Molecular biology, Software, Software

## Abstract

Biomarkers are crucial in aiding in disease diagnosis, prognosis, and treatment selection. Machine learning (ML) has emerged as an effective tool for identifying novel biomarkers and enhancing predictive modelling. However, sex-based bias in ML algorithms remains a concern. This study developed a supervised ML model to predict nine common clinical biomarkers, including triglycerides, BMI, waist circumference, systolic blood pressure, blood glucose, uric acid, urinary albumin-to-creatinine ratio, high-density lipoproteins, and albuminuria. The model’s predictions were within 5–10% error of actual values. For predictions within 10% error, the top performing models were waist circumference, albuminuria, BMI, blood glucose and systolic blood pressure, with males scoring higher than females, followed by the combined data set containing sex as an input feature and the combined data without sex as an input feature performing the poorest. This study highlighted the benefits of stratifying data according to sex for ML based models.

## Introduction

The term “biomarker” refers to any medical signal that can be precisely measured and provides valuable insights into an individual’s medical state^1,2^. In healthcare, biomarkers are crucial for diverse aspects of patient care, playing pivotal roles in disease diagnosis, progression prediction, and treatment selection and monitoring.^3,4^. Due to the intricate interactions of physiological systems, biomarkers often exhibit correlations with each other, enabling more robust diagnostic and prognostic evaluations. Predictive biomarkers are particularly valuable as they facilitate the development of personalised therapeutic approaches and pre-emptive interventions^5^. The increasing focus on clinical trials driven by biomarker research aims at optimising disease management through personalised healthcare strategies such as screening and risk assessments^4,6,7^.

Recently, machine learning (ML) techniques have gained attention as effective tools to uncover novel biomarkers^8–10^. ML algorithms are sensitive to high quality data and utilise a range of statistical, probabilistic, and optimisation techniques, drawing insights from prior knowledge to discern valuable patterns within vast, unorganised, and intricate datasets^11^. Big data also offers insight into relationships between various biomarkers and diseases, creating novel opportunities for predictive modelling in disease risk predictions^12^. By combining ML and extensive data sets one creates an opportunity for more accurate and reliable biomarker predictions.

In spite of the growing success of these ML methodologies, bias in biochemical algorithms is an overlooked issue, with one of them being sex based^13^. Straw and Wu examined the ML predictions around liver disease and found that although the various studies and industries were able to predict liver disease in patients >70%, with optimisations done on ML models and features selected, they all failed to examine the effect that biological sex differences had on the ML prediction capability. Their study showed sex disparity in model performance for algorithms built from a commonly used liver disease dataset. They were also able to show how biochemical algorithms may reinforce and exacerbate existing healthcare inequalities^13^. Wang et al. conducted a ML study exploring the effect of sex bias in prognosis of lung cancers. Their conclusion was that more researchers in the cancer field are considering the effect of sex bias on cancers as females are found to have a better prognosis than males. They also state the necessity of developing sex-specific diagnosis and prognosis models^14^. Tokodi et al. considered mortality predictors among patients undergoing cardiac resynchronization therapy. Their in-depth analysis of features showed a marked sex difference in mortality predictors^15^.

Taking into consideration that the exploration of biomarkers in healthcare, particularly through the lens of ML techniques, presents a promising avenue for revolutionising personalised medicine. It is imperative to address and mitigate biases within these algorithms, particularly concerning sex-based disparities, as highlighted by recent studies. Failure to account for such biases not only undermines the accuracy of predictive models but also perpetuates healthcare inequalities. Hence, the objective of this study was to develop accurate independent biomarker prediction models that account for sex-specific differences. To validate the accuracy of these predictions, standard ML evaluation techniques and diverse cross-validation statistical approaches were employed, thus affirming the strength and predictive capacity of the models.

## Results

### Clinical factors for the 1199 participants in the cohort

The descriptive statistics for the cohort were summarised in (Table 1). For each, the mean value was accompanied by the standard deviation in parentheses for continuous data and percentage for non-continuous data, providing an indication of the variability within the subgroup. The cohort exhibited a balanced sex distribution, encompassing diverse racial backgrounds and displaying homogeneity in age group representation. Notably, there was a higher prevalence of smokers within the male subgroup when compared to the female counterpart.

**Table 1:** Clinical factors and demographic statistics and counts for the N = 1199 participants in the NHANES cohort

Categories	Female	Male
***n*** = **1199**	604 (50.38%)	595 (49.62%)
Race *n* (%)	Asian: 99 (16.39) Black: 154 (25.50) Hispanic: 65 (10.76) MexAmerican: 47 (7.78) Other:8 (1.32) White:231 (38.25)	Asian: 90 (15.13) Black: 117 (19.66) Hispanic: 66 (11.09) MexAmerican: 66 (11.09) Other: 15 (2.52) White:241 (40.50)
**Married** *n* **(%)**	310 (51.32)	351 (58.99)
**Age (years)**	45.59 (±15.99)	45.10 (±16.05)
**Waist Circumference (cm)**	93.47 (±14.20)	97.11 (±13.62)
**BMI**^**a**^ **(kg/m²)**	27.87 (±5.85)	27.39 (±4.82)
**Albuminuria (mg/L)**	4.22 (±0.28)	4.40 (±0.29)
**UrAlbCr**^a^ **(mg/g)**	8.14 (±4.55)	6.13 (±4.07)
**Uric Acid (mmol/L)**	0.17 (±0.04)	0.21 (±0.04)
**Blood Glucose (mmol/L)**	5.37 (±0.62)	5.55 (±0.58)
**HDL**^**a**^ **(mmol/L)**	1.51 (±0.32)	1.28 (±0.28)
**Triglycerides (mmol/L)**	1.13 (±0.50)	1.25 (±0.53)
**Systolic BP**^**a**^ **(mmHg)**	116.59 (±15.15)	121.84 (±13.49)
**Smokers** *n* **(%)**	179 (29.64)	292 (49.08)

Values indicated in brackets are the standard deviation for continuous data and percentage for non-continuous data.

^a^*BMI* Body Mass Index, *UrAlbCr* urinary albumin-to-creatinine ratio, *HDL* High-Density Lipoprotein, *BP* Blood Pressure.

#### Patterns in data variance

**Fig. 1:**
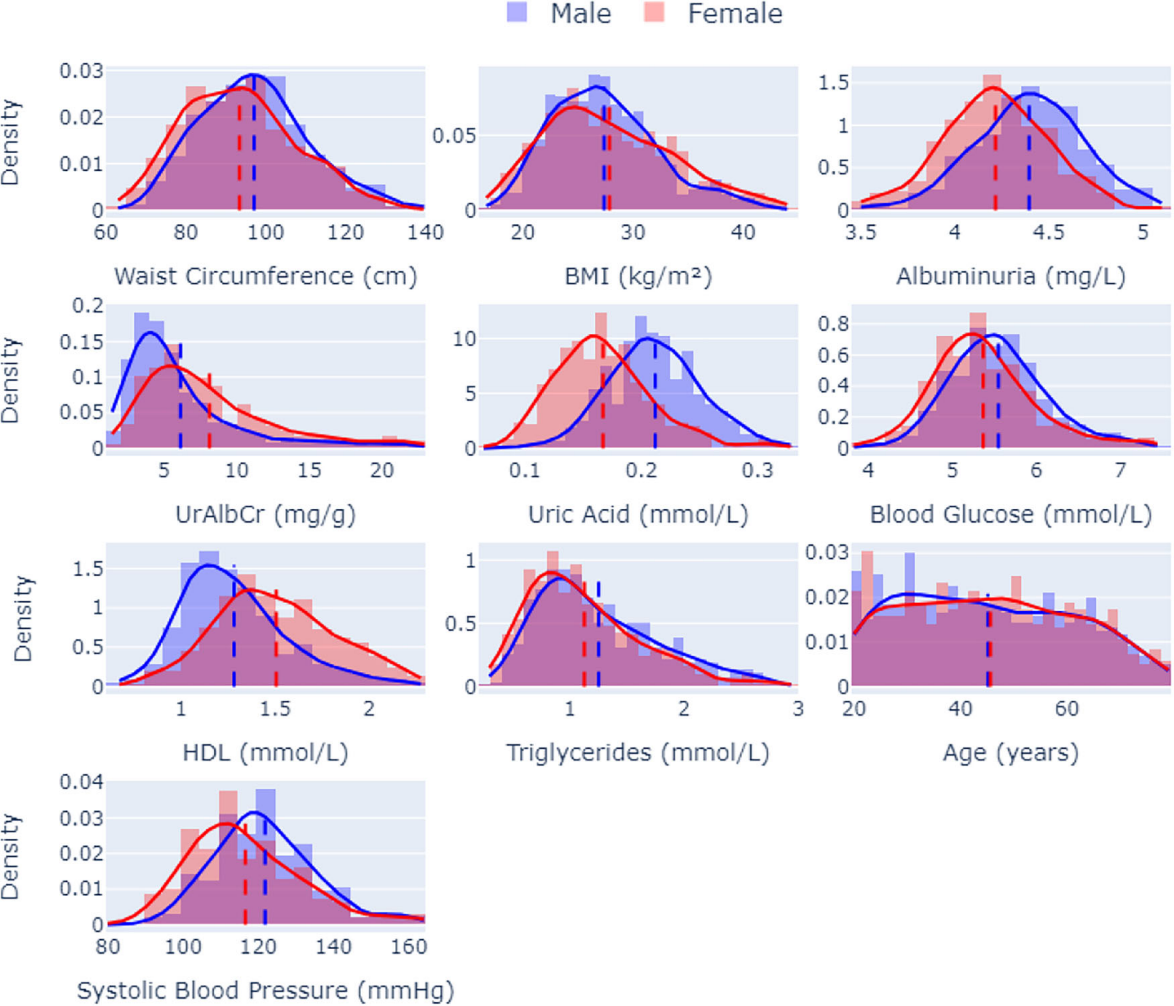
Distribution analysis of the NHANES data used in model training. Graphs showing the density distribution of training features: stratified according to sex.

Analysis was done on the data to explore the variance in the data distribution for both male and female subgroups. Figure 1 shows the density distribution across the various biomarkers, whereas Table 2 shows the statistical analysis done to determine the significant differences between mean and variances according to features between male and female groups.

**Table 2:** Summary of comparative analysis of various health biomarkers between male and female subgroups with respect to variance and mean values

Biomarker	Levene’s *p*-value (σ^2^)	Unpaired Sample t-Test	^a^U-Statistic	^b^Mann-Whitney U-Test
**Waist Circumference (cm)**	0.185	< 0.05	2.07e + 05	< 0.05
**BMI (kg/m²)**	< 0.05	0.121	1.75e + 05	0.406
**Albuminuria (mg/L)**	0.516	< 0.05	2.43e + 05	< 0.05
**UrAlbCr (mg/g)**	< 0.05	< 0.05	1.20e + 05	< 0.05
**Uric Acid (mmol/L)**	0.665	< 0.05	2.85e + 05	< 0.05
**Blood Glucose (mmol/L)**	0.436	< 0.05	2.15e + 05	< 0.05
**HDL (mmol/L)**	< 0.05	< 0.05	1.04e + 05	< 0.05
**Triglycerides (mmol/L)**	0.060	< 0.05	2.05e + 05	< 0.05
**Age (years)**	0.728	0.596	1.76e + 05	0.579
**Systolic Blood Pressure (mmHg)**	< 0.05	< 0.05	2.22e + 05	< 0.05

^a^The U-Statistic represents the computed value from the test, which is utilised to determine the p-value. In p-values below 0.05, indicating statistically significant differences between sexes for each biomarker.

^b^The Mann-Whitney U-Test was used to evaluate if there are significant differences in biomarker values between sexes. As a non-parametric test for independent samples, it does not assume normal distribution.

Density distributions of various training features across sex groups (male and female) for a dataset. Each subplot shows the distribution of a specific feature, such as Waist Circumference, BMI, Albuminuria, UrAlbCr, Uric Acid, Blood Glucose, HDL, Triglycerides, Age, and Systolic Blood Pressure, with density curves overlaid for males (in blue) and females (in red). The shaded areas represent the density distribution, and vertical dashed lines indicate the mean value for each sex.

The results revealed significant differences in both variance and mean values for certain biomarkers. Specifically, systolic blood pressure (mmHg), HDL cholesterol (mmol/L), and Urinary Albumin-to-Creatinine Ratio (UrAlbCr, mg/g) demonstrated notable differences between the sexes. For systolic blood pressure and HDL cholesterol, Levene’s test indicated significant variance differences, suggesting a broader range of values in one sex, which may reflect greater variability or differing influences on these biomarkers within that sex. Similarly, UrAlbCr showed significant differences in both variance and mean values, highlighting pronounced differences in biomarker expression between males and females.

In contrast, for biomarkers such as waist circumference (cm), albuminuria (mg/L), uric acid (mmol/L), blood glucose (mmol/L), and triglycerides (mmol/L), no significant variance-based differences were observed between sexes. However, the mean values for these biomarkers were significantly different, as evidenced by the Unpaired Sample t-Test and Mann-Whitney U-Test.

**Fig. 2:**
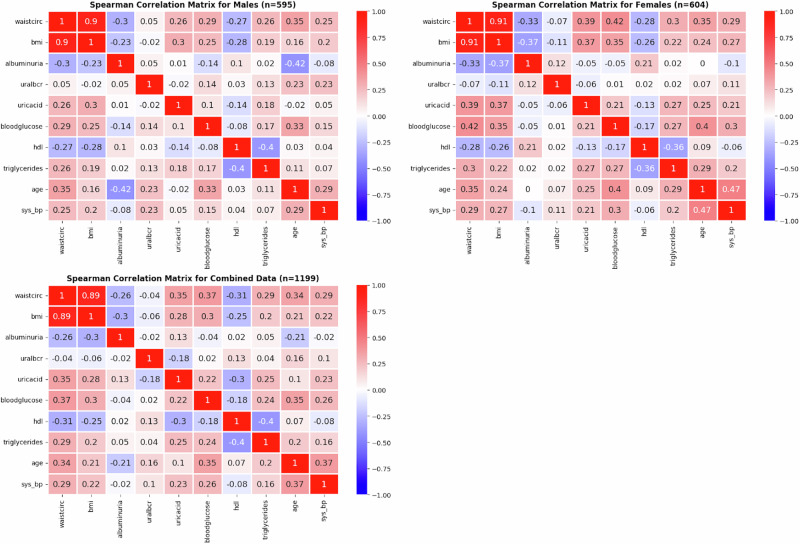
Spearman correlation for the model features: Each matrix has been stratified according to sex, with the last matrix indicating the male and female data being combined.

Figure 2 shows the spearman correlations in each of the male, female and combined data groups. This initial pairwise Spearman correlation analysis was conducted to explore the relationships among biomarkers and to support the feature selection process in this study. As shown in Fig. 2, the results revealed complex co-dependencies, which are more likely to emerge in multivariate, nonlinear, or higher-dimensional analyses. A notable exception was the strong positive correlation between BMI and waist circumference (r = 0.9) in both male and female subgroups. In contrast, most other biomarkers exhibited weak, positive or negative monotonic relationships. This suggests that while Spearman correlation captures pairwise monotonic associations, it may not fully account for the complex multivariate interactions essential for robust predictions.

The results also showed that the Spearman correlations for male and female subgroups were notably similar to the combined group, though some biomarker relationships differed when comparing the three. While the correlation between BMI and waist circumference remained consistently strong across all groups (*r* = 0.9), other biomarkers revealed sex-specific differences. For example, albuminuria had a stronger negative correlation with BMI in females (*r* = −0.37) compared to males (*r* = −0.23), with the combined group (*r* = −0.3) falling between the two, suggesting a more pronounced inverse relationship in females.

Additionally, age and systolic blood pressure demonstrated a more significant correlation in females (*r* = 0.47) than in males (r = 0.29), while the combined group showed a moderate positive correlation (*r* = 0.37), indicating that age-related increases in blood pressure are more pronounced in females. Similarly, HDL and triglycerides exhibited a slightly stronger inverse relationship in males (*r* = −0.4) compared to females (*r* = −0.36), with the combined group mirroring this relationship (*r* = −0.4).

Collectively, these results highlight that while combined data provides an overall view, it can obscure subtle but important sex-specific differences in biomarker interactions. These findings suggested that separate analyses for males and females may offer more precise insights, particularly when developing predictive models for healthcare interventions.

Spearman’s rank correlation coefficients were computed for 10 feature pairs separately for male, female and combined groups. Results were visualised with positive correlations highlighted in red and negative relationships in blue.

### Biomarker prediction optimisation including feature selection (RFECV)

Despite the weak pairwise correlations, the predictive value was captured when analysed as part of a multivariate framework^16–19^. To this end, Recursive Feature Elimination with Cross-Validation (RFECV) was applied to the full set of 14 biomarkers, aiming to identify the most relevant features for training individual models. This approach enhanced prediction accuracy, minimised error, and optimised the number of features required for an ideal validation score.

Certain demographic factors, such as race and marital status, were excluded from the feature set due to their association with model inaccuracies. The remaining 12 features were retained for further analysis.

Table 3 shows the feature sets for predicting various biomarker targets within a multivariate framework, applied to female-specific, male-specific, and combined datasets. For instance, the biomarker target “Albuminuria” was best predicted in females using body mass index (BMI), high-density lipoprotein (HDL), waist circumference, triglycerides, uric acid, and urine albumin-to-creatinine ratio (UrAlbCr). In contrast, the predictors for males included age, waist circumference, triglycerides, BMI, HDL, smoker status, and UrAlbCr as input variables. This pattern of varying optimal markers across datasets was observed across all listed biomarkers. Interestingly, biomarker targets in the combined datasets required more features hinting towards data pattern relationship complexity.

**Table 3:** Features selected for biomarker optimisation

Biomarker Target	Optimal Features for Females	Optimal Features for Males	Optimal Features for Combined Data	Optimal Features for Combined Data with sex as an input feature
**Albuminuria**	BMI, HDL, Waist circumference, Triglycerides, Uric acid, UrAlbCr	Age, Waist circumference, Triglycerides, BMI, HDL, Smoker, UrAlbCr	BMI, Age, Uric acid, Triglycerides, HDL, Systolic blood pressure, Blood glucose, Waist Circumference, Smoker.	Sex, BMI, Age, HDL, Triglycerides, Waist circumference, Uric acid, UrAlbCr, Blood glucose
**Blood glucose**	Age, Waist circumference, Systolic blood pressure, HDL	Age, BMI, UrAlbCr, Triglycerides	Age, Waist circumference, Systolic blood pressure,BMI, HDL, Uric acid, Triglycerides, Albuminuria	Age, BMI, Waist circumference, Systolic blood pressure, HDL, Triglycerides, Sex, Albuminuria, Uric acid
**BMI**	Waist circumference, UrAlbCr, Age, Triglycerides, Albuminuria, Uric acid, Blood glucose, Systolic blood pressure, HDL, Smoker	Waist circumference, Age	Waist Circumference, Age, Albuminuria, Triglycerides, UrAlbCr, Uric acid, Blood glucose, Systolic blood pressure	Waist circumference, Sex, Age, UrAlbCr, Triglycerides, Uric acid, Smoker, Albuminuria, Blood glucose, Systolic blood pressure, HDL
**HDL**	Triglycerides, Age, Albuminuria, Waist Circumference, Blood glucose	Triglycerides, Waist circumference, Age, Albuminuria, Systolic blood pressure	Waist circumference, Triglycerides, Age, BMI, Uric Acid, Albuminuria, Blood glucose, UrAlbCr	Triglycerides, Sex, Age, Albuminuria, Waist circumference, Blood glucose
**Systolic blood pressure**	Age, BMI, Blood glucose, UrAlbCr	UrAlbCr, Age, Waist circumference, HDL	Age, Uric acid, Waist circumference, UrAlbCr, Triglycerides, Blood glucose, HDL, BMI, Albuminuria	Age, UrAlbCr, Waist circumference, BMI, Blood glucose, Triglycerides, HDL, Uric acid, Albuminuria, Sex
**Triglycerides**	HDL, Waist circumference, Age, BMI, Uric acid, Albuminuria	Waist circumference, HDL, BMI, Albuminuria, Uric acid, Blood glucose, UrAlbCr, Age	HDL, Waist circumference, Age, Albuminuria, BMI, Uric Acid, Blood glucose, Smoker, UrAlbCr	HDL, Waist circumference, BMI, Sex, Albuminuria, Age, Uric acid, Smoker, Blood glucose, Systolic blood pressure, UrAlbCr
**UrAlbCr**	Systolic blood pressure, Albuminuria, Waist circumference	Age, Systolic blood pressure, Triglycerides, Blood glucose, BMI, Waist circumference, Uric acid, HDL, Albuminuria, Smoker	Uric acid, Age, Triglycerides, BMI, HDL, Systolic blood pressure, Waist circumference	Age, Uric acid, Sex, BMI, Systolic blood pressure, Triglycerides, Blood glucose, Waist circumference, Albuminuria, HDL
**Uric acid**	BMI, Triglycerides, Albuminuria, Age, Waist circumference, Blood glucose	BMI, Triglycerides	Waist circumference, Albuminuria, Systolic blood pressure, HDL, UrAlbCr, Blood glucose, Triglycerides	Sex, BMI, Waist circumference, Triglycerides, Albuminuria, Systolic blood pressure, UrAlbCr, Blood glucose, Smoker
**Waist circumference**	BMI, Age, Smoker, Triglycerides, Blood glucose, HDL, Albuminuria, Systolic blood pressure	BMI, Age, Triglycerides, HDL	BMI, Age, Uric acid, HDL, Smoker, Triglycerides	BMI, Age, Sex, Triglycerides, Smoker, HDL

^a^*BMI* Body Mass Index, *UrAlbCr* urinary albumin-to-creatinine ratio, *HDL* High-Density Lipoprotein.

**Fig. 3:**
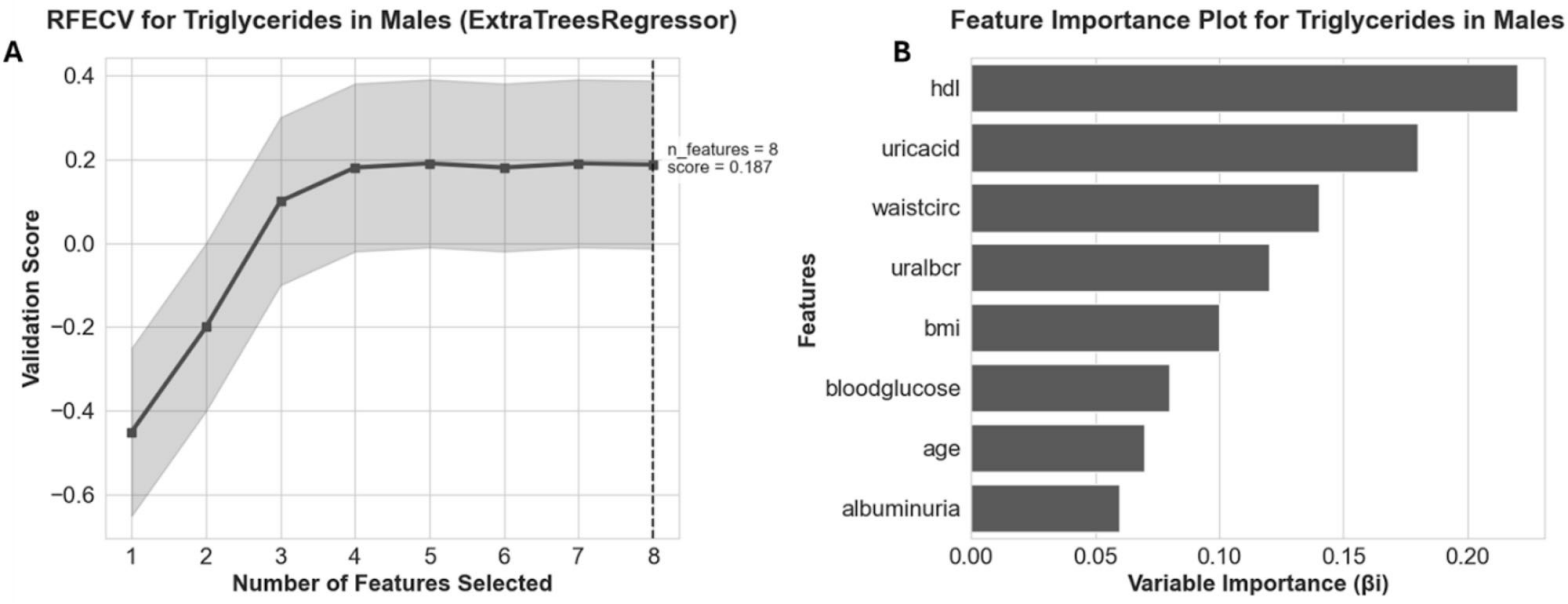
Example of RFECV and variable importance methodology used for each model target.

**Fig. 4:**
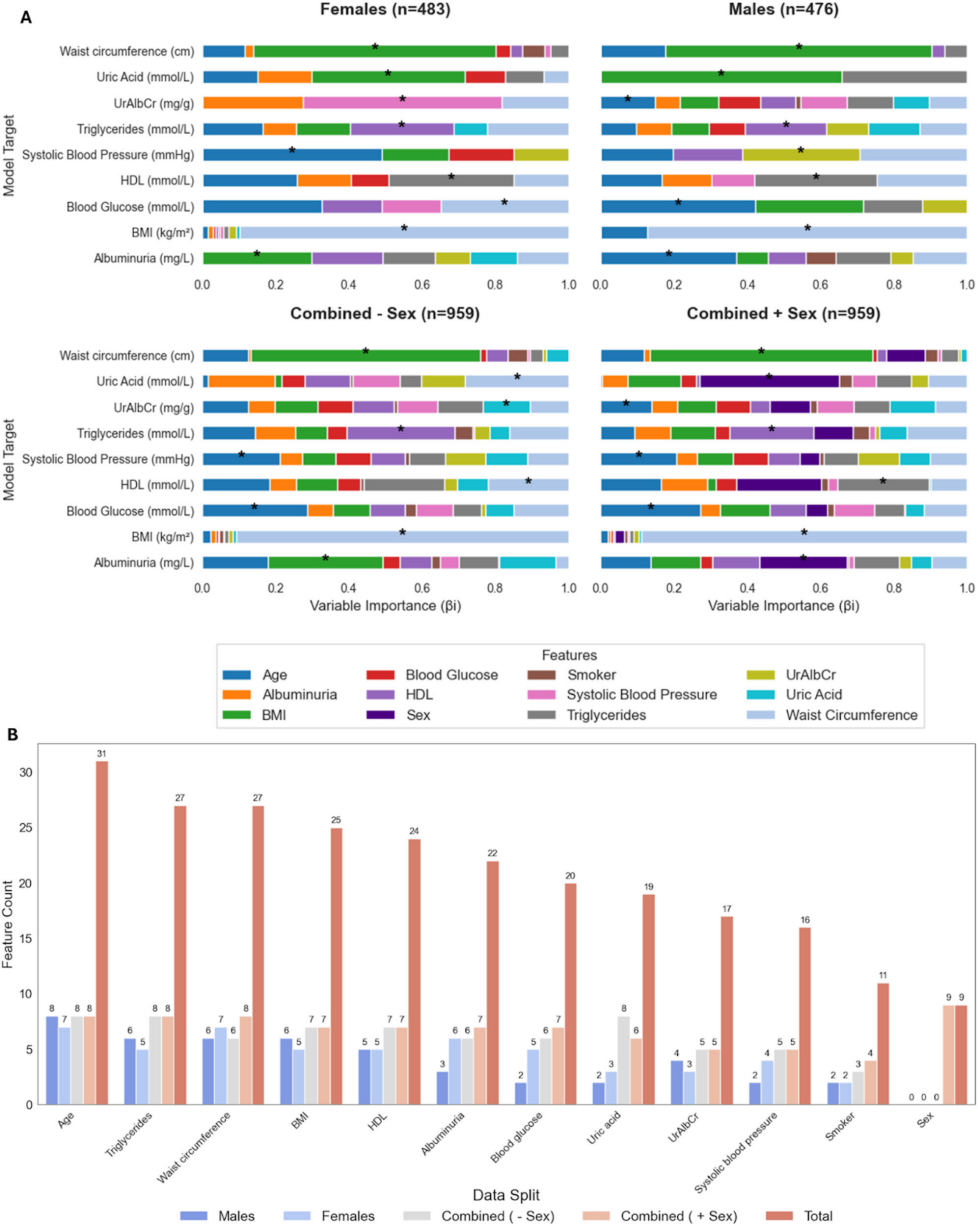
Demonstrates the impact of variable importance (βi) and variable importance for optimising the machine learning model to predict triglyceride levels in males (as an example). **A** Depicts model performance (validation score) across different feature counts, highlighting the optimal number of features. **B** Illustrates variable importance of features (βi) used in the prediction.

The findings in Table 2 further revealed sex-specific variations in biomarkers, which guided the feature selection process outlined in Table 3 and illustrated in Fig. 3. This process demonstrated that sex-based variability substantially impacted feature selection by altering coefficient importance (*β*_i_), potentially explaining the observed differences in selected features presented in Table 3^17–19^. Optimal feature selection, recorded in Table 3, was determined by analysing cross-validation scores relative to variable importance, as demonstrated in Fig. 3. Specifically, Fig. 3A identified the optimal number of features for the model, while Fig. 3B highlighted the most influential features (Variable Importance) and the corresponding (*β*_i_) values. This methodology^19^ ensured the selection of only the most significant features and the development of the most effective model, addressing sex-specific influences and reducing the risk of overfitting, as further detailed in Fig. 4.

Figure 4A presents a side-by-side comparison of variable importance plots for females, males, and combined groups. These plots illustrate the standardised and weighted βi values of key features, ranked in order of their contribution to optimising each biomarker model (Model Target).

**A** Variable Importance (βi) of Predictors for Model Targets, Grouped by Sex and Combined Data in Feature Analysis. Each panel: one for females, one for males, one for the combined data, and the combined data with sex as the input feature presents horizontal stacked bar charts illustrating the importance of each predictor. Asterisks (*) highlight the most significant predictor for each model target for each group. (**B**) Displays the frequency count of each biomarker used as a feature in the optimisation process across male, female, and combined subgroups.

In Fig. 4A, for the female subgroup, waist circumference was the most significant contributor to the BMI model target, age to systolic blood pressure and blood glucose, HDL to triglycerides, triglycerides to HDL, and systolic blood pressure to UrAlbCr. Notably, BMI was the top contributor to three biomarkers: albuminuria, uric acid, and waist circumference. In contrast, the male subgroup showed a different pattern. Waist circumference contributed to both the BMI and triglyceride models, age to blood glucose, albuminuria, and UrAlbCr, BMI to uric acid and waist circumference, UrAlbCr to systolic blood pressure, and triglycerides to HDL. In the combined data, the pattern seen was that the significant contributor for each model target was either the same as the male or female and sometimes all 4 data sets contained the same variable, however, UrAlbCr was the only biomarker where all 4 differed entirely.

In Fig. 4B, age was the most frequently selected feature for both male and female groups in the ML models. For blood glucose, the analysis showed a higher frequency count in females compared to males. The analysis of feature selection across sex revealed distinct trends in feature frequency: In males, features having higher counts than the females were BMI, triglycerides and UrAlbCr. In females, waist circumference and albuminuria clustered together, while BMI, triglycerides, blood glucose, and HDL also tended to cluster. In the combined groups, the ML required almost all the features as inputs, in order to make more accurate predictions of the biomarkers compared to the sex-stratified groups.

The results in Table 4, showed the best ML model and corresponding metrics for the predictions of the various biomarkers for the four groups. A comparison table showing all 19 models evaluated against each other for all 9 biomarkers for the female and male subgroups can be found in the supplementary (Supplementary Table 1). The ML model that performed the best based on the data was chosen irrespective of the sex for the remaining processes.

**Table 4:** Best performing models for the various biomarkers in both male and female subgroups as well as combined data and combined data with sex as an input feature

	Prediction Target	Model	MAE (Unit)	MSE (Unit²)	RMSE (Unit)	R²	RMSLE	MAPE
**Female**	**Waist circumference (cm)**	Bayesian Ridge	4.95 cm	40.55 cm²	6.37 cm	0.8	0.07	0.05
	**BMI (kg/m²)**	Gradient Boosting Regressor	2.05 kg/m²	7.54 kg/m²	2.75 kg/m²	0.77	0.09	0.07
	**UrAlbCr (mg/g)**	Linear Regression	3.11 mg/g	16.51 mg/g²	4.06 mg/g	-0.01	0.46	0.53
	**Uric Acid (mmol/L)**	Huber Regressor	0.03 mmol/L	0.00 mmol/L²	0.04 mmol/L	0.11	0.04	0.19
	**Blood Glucose (mmol/L)**	Linear Regression	0.47 mmol/L	0.36 mmol/L²	0.60 mmol/L	0.17	0.09	0.09
	**HDL (mmol/L)**	Bayesian Ridge	0.21 mmol/L	0.07 mmol/L²	0.26 mmol/L	0.23	0.1	0.15
	**Triglycerides (mmol/L)**	Linear Regression	0.33 mmol/L	0.21 mmol/L²	0.46 mmol/L	0.21	0.2	0.33
	**Albuminuria (mg/L)**	Bayesian Ridge	0.19 mg/L	0.06 mg/L²	0.24 mg/L	0.2	0.05	0.05
	**Systolic Blood Pressure (mmHg)**	Huber Regressor	10.37 mmHg	175.94 mmHg²	13.26 mmHg	0.24	0.11	0.09
**Male**	**Waist circumference (cm)**	Huber Regressor	3.90 cm	25.39 cm²	5.04 cm	0.86	0.05	0.04
	**BMI (kg/m²)**	Ridge	1.43 kg/m²	3.15 kg/m²	1.77 kg/m²	0.86	0.06	0.05
	**UrAlbCr (mg/g)**	Random Forest Regressor	2.84 mg/g	19.30 mg/g²	4.39 mg/g	0	0.49	0.46
	**Uric Acid (mmol/L)**	Bayesian Ridge	0.03 mmol/L	0.00 mmol/L²	0.04 mmol/L	0.12	0.04	0.17
	**Blood Glucose (mmol/L)**	Huber Regressor	0.40 mmol/L	0.28 mmol/L²	0.53 mmol/L	0.14	0.08	0.07
	**HDL (mmol/L)**	Linear Regression	0.20 mmol/L	0.07 mmol/L²	0.27 mmol/L	0.14	0.11	0.15
	**Triglycerides (mmol/L)**	ExtraTrees Regressor	0.41 mmol/L	0.26 mmol/L²	0.51 mmol/L	0.13	0.21	0.34
	**Albuminuria (mg/L)**	Bayesian Ridge	0.21 mg/L	0.07 mg/L²	0.26 mg/L	0.14	0.05	0.05
	**Systolic Blood Pressure (mmHg)**	Huber Regressor	10.38 mmHg	176.90 mmHg²	13.30 mmHg	0.08	0.11	0.09
**Combined Data excluding sex as an input feature**	**Waist circumference (cm)**	Bayesian Ridge	4.16 cm	32.15 cm²	5.67 cm	0.83	0.06	0.04
	**BMI (kg/m²)**	Gradient Boosting Regressor	1.76 kg/m²	5.47 kg/m²	2.34 kg/m²	0.81	0.08	0.06
	**UrAlbCr (mg/g)**	Random Forest Regressor	2.85 mg/g	15.92 mg/g²	3.99 mg/g	0.058	0.46	0.50
	**Uric Acid (mmol/L)**	BayesianRidge	0.03 mmol/L	0.00 mmol/L²	0.04 mmol/L	0.23	0.03	0.16
	**Blood Glucose (mmol/L)**	Huber Regressor	0.43 mmol/L	0.30 mmol/L²	0.55 mmol/L	0.21	0.08	0.08
	**HDL (mmol/L)**	LinearRegression	0.21 mmol/L	0.07 mmol/L²	0.27 mmol/L	0.27	0.11	0.16
	**Triglycerides (mmol/L)**	LinearRegression	0.35 mmol/L	0.22 mmol/L²	0.47 mmol/L	0.20	0.20	0.35
	**Albuminuria (mg/L)**	Ridge	0.21 mg/L	0.07 mg/L²	0.27 mg/L	0.15	0.05	0.05
	**Systolic Blood Pressure (mmHg)**	Random Forest Regressor	10.77 mmHg	198.82 mmHg²	14.10 mmHg	0.13	0.11	0.09
**Combined Data including sex as an input feature**	**Waist circumference (cm)**	Bayesian Ridge	3.98 cm	28.10 cm²	5.30 cm	0.85	0.05	0.04
	**BMI (kg/m²)**	Gradient Boosting Regressor	1.62 kg/m²	4.53 kg/m²	2.13 kg/m²	0.85	0.07	0.06
	**UrAlbCr (mg/g)**	AdaBoost Regressor	2.78 mg/g	15.32 mg/g²	3.91 mg/g	0.09	0.45	0.51
	**Uric Acid (mmol/L)**	Bayesian Ridge	0.03 mmol/L	0.00 mmol/L²	0.04 mmol/L	0.35	0.03	0.15
	**Blood Glucose (mmol/L)**	Bayesian Ridge	0.43 mmol/L	0.30 mmol/L²	0.55 mmol/L	0.21	0.08	0.08
	**HDL (mmol/L)**	Linear Regression	0.20 mmol/L	0.07 mmol/L²	0.26 mmol/L	0.34	0.11	0.15
	**Triglycerides (mmol/L)**	Linear Regression	0.35 mmol/L	0.21 mmol/L²	0.46 mmol/L	0.23	0.20	0.34
	**Albuminuria (mg/L)**	Bayesian Ridge	0.21 mg/L	0.07 mg/L²	0.27 mg/L	0.19	0.05	0.05
	**Systolic Blood Pressure (mmHg)**	Random Forest Regressor	10.56 mmHg	194.87 mmHg²	13.96 mmHg	0.15	0.11	0.09

When examining Table 4, the top performing models were waist circumference, BMI, systolic blood pressure, blood glucose and albuminuria. The overall trend seen in these metrics were that male and female subgroups, had lower values than the combined groups indicating higher performances with the following evidence:

#### Waist circumference

The male subgroup had lower MAE, MSE and higher R² values than the female group, indicating a better prediction accuracy for males. Both combined models fell between the subgroups, hinting towards an averaging out of the sex-specific differences. The combined plus sex as the input variable had lower values than the combined data without sex indicating a more accurate model.

Female: Model = Bayesian Ridge, MAE = 4.95 cm, MSE = 40.55 cm², RMSE = 6.37 cm, R² = 0.8.

Male: Model = Huber Regressor, MAE = 3.90 cm, MSE = 25.39 cm², RMSE = 5.04 cm, R² = 0.86

Combined: Model = Bayesian Ridge, MAE = 4.16 cm, MSE = 32.15 cm², RMSE = 5.67 cm, R² = 0.83

Combined + sex = Bayesian Ridge, MAE = 3.98 cm, MSE = 28.10 cm², RMSE = 5.30 cm, R² = 0.85

#### BMI

The results in this group followed the same pattern as seen with the waist circumference for all 4 data sets.

Female: Model = Gradient Boosting Regressor, MAE = 2.05 kg/m², MSE = 7.54 kg/m², RMSE = 2.75 kg/m², R² = 0.77

Male: Model = Ridge, MAE = 1.43 kg/m², MSE = 3.15 kg/m², RMSE = 1.77 kg/m², R² = 0.86

Combined: Model = Gradient Boosting Regressor MAE = 1.76 kg/m², MSE = 5.47 kg/m², RMSE = 2.34 kg/m², R² = 0.81

Combined + sex = Gradient Boosting Regressor, MAE = 1.62 kg/m², MSE = 4.53 kg/m², RMSE = 2.13 kg/m², R² = 0.85

#### Blood glucose

Although this model was able to predict glucose, there is room for improvement across all 4 groups. The results also explained only a small portion of the variability in the target variable, and its predictions had a noticeable average deviation from the actual values, with both of the combined models being slightly better. For this biomarker, it would appear that sex as an input feature had no influence on the validation metric results as the values were identical for both combined data sets.

Female: Model = Linear Regression, MAE = 0.47 mmol/L, MSE = 0.36 mmol/L², RMSE = 0.60 mmol/L, R² = 0.17

Male: Model = Huber Regressor, MAE = 0.40 mmol/L, MSE = 0.28 mmol/L², RMSE = 0.53 mmol/L, R² = 0.14

Combined: Model = Huber Regressor, MAE = 0.43 mmol/L, MSE = 0.30 mmol/L², RMSE = 0.55 mmol/L, R² = 0.21

Combined + sex: Model = Bayesian Ridge, MAE = 0.43 mmol/L, MSE = 0.30 mmol/L², RMSE = 0.55 mmol/L, R² = 0.21

#### Systolic blood pressure

For this biomarker the female subgroup’s prediction metrics were slightly better when compared with the male subgroup. Both combined models showed a slightly higher error which could be due to the inherent biological sex-differences in blood pressure patterns. These results indicated that the model was well-suited to predict values within the first, second, and third quartile ranges but could face challenges in accurately predicting values outside of these ranges.

Female: Model = Huber Regressor, MAE = 10.37 mmHg, MSE = 175.94 mmHg², RMSE = 13.26 mmHg, R² = 0.24

Male: Model = Huber Regressor, MAE = 10.38 mmHg, MSE = 176.90 mmHg², RMSE = 13.30 mmHg, R² = 0.08

Combined: Model = Random Forest Regressor, MAE = 10.77 mmHg, MSE = 198.82 mmHg², RMSE = 14.10 mmHg, R² = 0.13

Combined + sex: Model = Random Forest Regressor, MAE = 10.56 mmHg, MSE = 194.87 mmHg², RMSE = 13.96 mmHg, R² = 0.15

#### Albuminuria

For this biomarker the female subgroup performed slightly better than both the male and combined models in predicting albuminuria. These findings suggested that the models demonstrated strong predictive ability for the target variable with minimal errors and high accuracy overall. Interestingly the ML model chose Ridge for the combined data whereas the male, female and combined with sex groups had the same Bayesian Ridge Model.

Female: Model = Bayesian Ridge, MAE = 0.19 mg/L, MSE = 0.06 mg/L², RMSE = 0.24 mg/L, R² = 0.20

Male: Model = Bayesian Ridge, MAE = 0.21 mg/L, MSE = 0.07 mg/L², RMSE = 0.26 mg/L, R² = 0.14

Combined: Model = Ridge, MAE = 0.21 mg/L, MSE = 0.07 mg/L², RMSE = 0.27 mg/L, R² = 0.15

Combined + sex: Model = Bayesian Ridge, MAE = 0.21 mg/L, MSE = 0.07 mg/L², RMSE = 0.27 mg/L, R² = 0.19

**Fig. 5:**
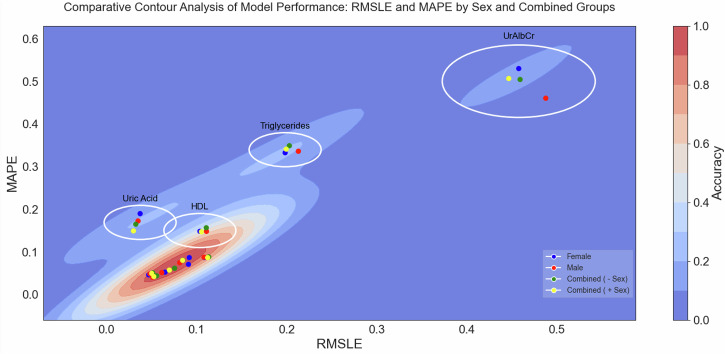
Comparative contour analysis of model performance: RMSLE and MAPE by sex and combined groups.

The contour plot in Fig. 5. provided a visual representation of the relationship between two key performance metrics, Root Mean Squared Logarithmic Error (RMSLE) and Mean Absolute Percentage Error (MAPE) from Table 4, for the biomarker targets for all 4 data groups. Both metrics serve as indicators of model accuracy, where lower values signify better performance. The clustering of data points in the lower regions of both axes indicated that the majority of models exhibited low error rates across both metrics. Specifically: RMSLE captured the logarithmic differences between predicted and actual values, with a focus on penalising large errors more heavily. MAPE reflected the percentage error between predicted and actual values, offering insight towards a measure of accuracy. The proximity of most data points to the lower left corner of the contour plot highlighted the overall robustness of the models, indicating that both male and female models performed comparably well with minimal prediction errors. This trend, coupled with the regression coefficients, suggested that the models maintained reliable performance across different error metrics.

Contour plot of RMSLE versus MAPE, demonstrating the performance of all the biomarker models developed in the study. The data points predominantly cluster in the lower left regions of both RMSLE and MAPE axes, indicating minimal prediction errors across all 4 model targets. This concentration suggested consistent accuracy and low variance in model performance (Refer to Table 6).

Figure 5 showed a significant positive correlation between MAPE and RMSLE for both male (*r* = 0.91), female (*r* = 0.92), combined without sex as an input variable (*r* = 0.92), and combined with the sex input variable (0.93) models. The high correlation suggested that these error metrics were closely aligned, such that a reduction on one tended to be accompanied by a reduction in the other.

From this research we concluded that models such as waist circumference =β0 + β1⋅BMI + β2⋅Sex+β3⋅(BMI⋅Sex) could capture differences between sexes within a unified framework as it is arguably conserved as a function of BMI and sex. However, these types of models assumed that the relationship between predictors and outcomes were fundamentally similar for both sexes.

This research assumed disparate differences existed and therefore involved separate models for males and females, acknowledging that physiological and biomarker differences may require distinct model structures. For instance, Table 4 showed that the optimal model architecture for predicting various model targets in males may not align with the features or interactions identified in females. In support of this, the tailored feature selection Fig. 5A, B, highlighted the influence of variable importance (βi) and the effect of weighting on the optimisation of models (Table 3) when developing distinct models for each sex. This approach allowed for precise tailoring of feature selection based on each group’s specific biological or physiological characteristics. Such customisation was not achievable with a general interaction model, where the same predictors were applied uniformly to both sexes. This assumption was also supported and tested by the comparison of sex-specific models against the same sex and opposite sex. What was found was that the models generally outperformed when tested within the same sex. There were instances where a female-trained model generalized well on male data and vice versa, however this was not the common trend. This suggests that while some features may be transferable across sexes, others are inherently sex-specific, influencing overall predictive accuracy. These results are shown in the supplementary (See Supplementary Figs. 1 and 2, along with Tables 2 and 3).

Figure 5 showed that the predictive power of the separate models for males and females remained even in the absence of model standardisation. This method, which avoided standardising models or features, allowed for the optimisation of each model target based on MAPE and RMSLE performance metrics specific to each subgroup. The contour plot analysis supported this conclusion, indicating that accuracy was preserved, with the majority of models falling within the 0.8 to 1 accuracy range and corresponding MAPE and RMSLE values between 0.05 and 0.1.

### Validation test of actual results vs predictive results

**Fig. 6:**
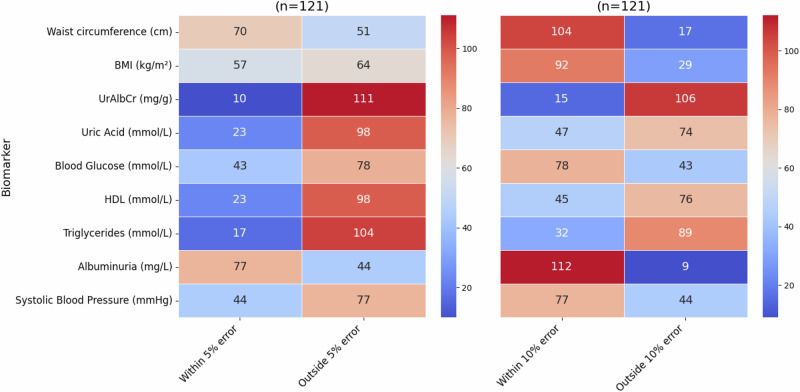
Validation test for females. The heatmap shows the number of individuals where the predicted value falls either within or outside of a 5% and 10% error of the actual value in the female subgroup (from the n = 121 test group).

**Fig. 7:**
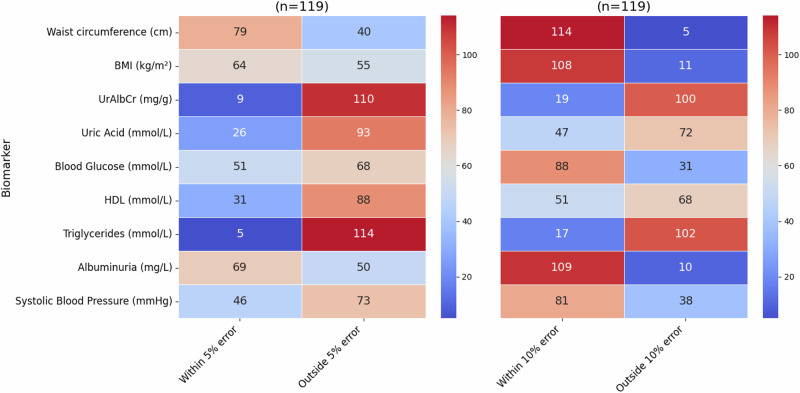
Validation Test for Males. The heatmap shows the number of individuals where the predicted value falls either within or outside of a 5% and 10% error of the actual value in the male subgroup (from the n = 119 test group).

**Fig. 8:**
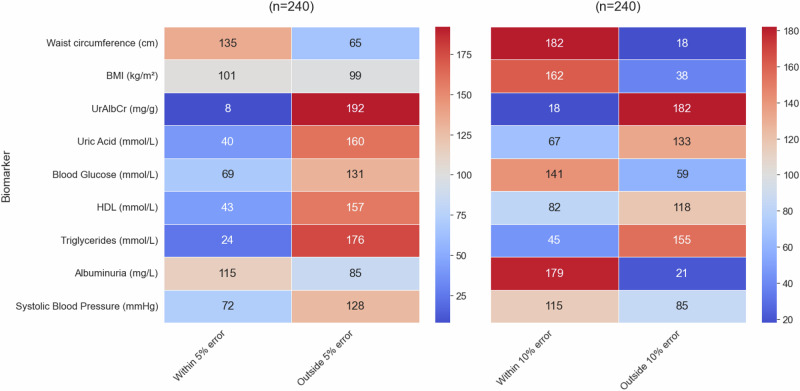
Validation test for combined - sex group (where sex has been removed an an input feature). The heatmap shows the number of individuals where the predicted value falls either within or outside of a 5% and 10% error of the actual value in the combined data group (from the n = 240 test group).

After creating and refining the prediction models, a validation test was performed on the hold out set (Test Data) for all four data groups for each of the biomarkers. To evaluate the predictive ability on the test set, the results were grouped according to “within a 5% and 10% error” respectfully. Heatmaps showing these results are displayed in Figs. 6–8 for the female and male subgroups, and the combined data. Higher values (shown in red) demonstrated effective predictions by the optimised ML model, while blue indicated ineffective predictions.

As seen in Fig. 6, the biomarkers that had the majority of individuals falling within the 10% error category were albuminuria, waist circumference, BMI, blood glucose, and systolic blood pressure. Of these biomarkers, the one consisting of the highest number of individuals was albuminuria with 93%, waist circumference with 86%, BMI with 76%, and the lowest two being blood glucose and systolic blood pressure with 64%. From a validation metrics point of view these were also the top performers with the exception of systolic blood pressure having a very high MSE value. The biomarker with the least number of individuals in the “within 10% error” category was UrAlbCr with 12%, followed by triglycerides (26%), HDL (37%), and uric acid (39%) respectively.

Despite a 10% error being acceptable for prediction purposes, in a clinical sense, that error could result in an individual falling incorrectly into an abnormal diagnostic range. For this reason a error of 5% was also evaluated and it can be seen that albuminuria resulted in 64% of the individuals falling within this range, followed by waist circumference (58%). UrAlbCr (8%) and triglycerides (14%) were the lowest two biomarkers respectively. Both uric acid and HDL had the same percentage (19%) of individuals for this category.

Heatmap showing the number of individuals where the predicted value falls either within or outside of a 5% and 10% error of the actual value in the male subgroup (from the *n* = 119 test group).

Upon examining Fig. 7, the male subgroup had a higher percentage of individuals falling within the “within the 10% error” category compared to the female group overall: waist circumference (96%), albuminuria (92%), BMI (91%), blood glucose (74%), and systolic blood pressure (68%) with HDL (43%), uric acid (39%), UrAlbCr (16%), and triglycerides (14%).

Within the 5% margin of error for the male subgroup, waist circumference exhibited the greatest number of individuals, followed by albuminuria. A similar pattern was noted in the female subgroup with these markers occurring in reverse order. BMI also had more than 50% of the individuals falling within this category which was different from the female group. Again, the lowest two biomarkers for this category were triglycerides and UrAlbCr, which was reversed in the female subgroup.

Heatmap showing the number of individuals where the predicted value falls either within or outside of a 5% and 10% error of the actual value in the combined data group with sex as an input feature (from the *n* = 240 test group).

When observing the validation results (Fig. 8) for the combined data most of the top predictions fell within the 10% error group. Waist circumference was the highest (76%), followed by albuminuria (75%), BMI (68%), blood glucose (59%), with systolic blood pressure (48%), HDL (34%), uric acid (28%), triglycerides (19%) and UrAlbCr (8%) falling below the 50% mark. Waist circumference was the only biomarker to have a prediction of above 50% within the 5% error range.

**Fig. 9:**
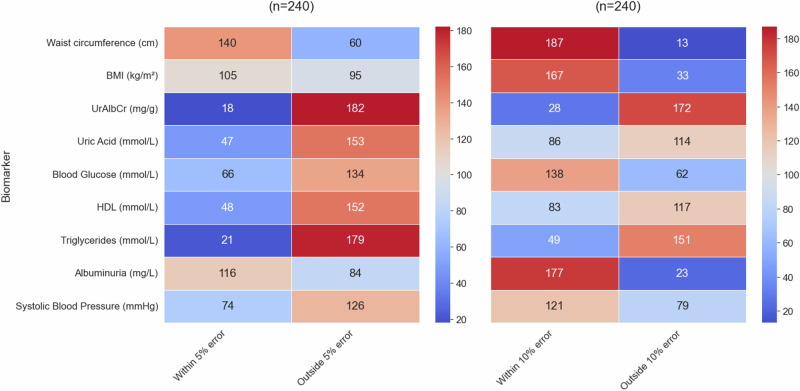
Validation test for combined + sex group (where sex is included as a feature). The heatmap shows the number of individuals where the predicted value falls either within or outside of a 5% and 10% error of the actual value in the combined data group (from the n = 240 test group).

The results seen in the validation results (Fig. 9) for the combined data containing the sex input feature, were very similar to the combined data set without it. The results were as follows in order of highest to lowest percentage for the within 10% error range were: Waist circumference (78%), albuminuria (74%), BMI (70%), blood glucose (58%), blood pressure (50%), uric acid (36%), HDL (35%), and triglycerides and UrAlbCr having 20%. The within 5% error group were very similar between the combined data and combined with sex input feature with the exception of UrAlbCr having 3% and 8% respectively.

**Fig. 10:**
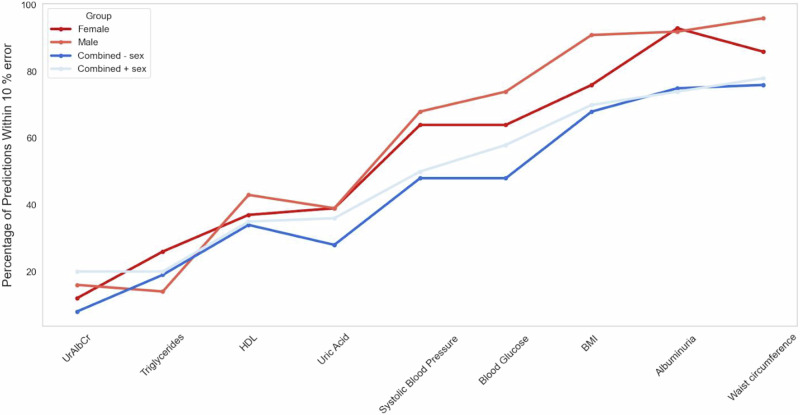
Group wise validation test results for prediction targets (Higher is better).

When comparing the results for the validation tests collectively (Fig. 10) waist circumference had the highest prediction within the 10% error for all data groups except the female subgroup, which was albuminuria. BMI was third on the list for all the data groups followed by blood glucose, and then blood pressure. The final 4 biomarkers were in different orders of prediction capability for all 4 groups. Overall the male subgroup data predicted better than the other data groups followed by the female subgroup, then combined with sex and finally the combined without sex data predicting the lowest. The divergence in the data e.g. between male and female subgroups for waist circumference, BMI and blood glucose indicated that these models were able to discriminate between data patterns more effectively (also seen in the clustering found in Fig. 5). The results seen in the validation test were consistent with the validation metrics determined in Table 4, Figs. 4 and 5.

## Discussion

This study highlighted the effectiveness of supervised machine learning (ML) in capturing both sex-specific subgroups and combined-sex multivariate relationships between biomarkers related to metabolic syndrome and cardiovascular health. Using the RFECV approach allowed for the identification of complex interactions among biomarkers that might be missed by traditional analytical methods. The findings underscored the importance of sex-specific models, as combining the data tended to average out results and obscure the unique biological variations between sexes. Although the models demonstrated strong validation scores, suggesting robustness, certain biomarkers—such as triglycerides, HDL, uric acid, and UrAlbCr—showed weaker predictive performance. This indicated a need for further refinement in model choice, hyperparameter tuning, and feature selection to improve accuracy for these markers. When examining the weaker performing markers with published literature these were the key similarities and differences in the methodologies;

When exploring uric acid; Sampa et al. were able to successfully predict uric acid using a similar approach to this study. In this study a RMSE of 0.04 mmol/L for both subgroups was obtained, which was similar to Sampa et al.’s RMSE of 0.03 mmol/L. A notable distinction between these two studies is that Sampa et al. utilised a set of 17 input features, comprising 12 core clinical measurements, 3 socio demographic attributes, and 2 dietary parameters. Of these, five features overlapped with those employed in the female subgroup of our study, namely sex, BMI, age, waist circumference, and blood glucose. In the male subgroup, only BMI coincided with the features employed by Sampa et al.^20^. The models they explored were Boosted Decision Tree Regression, Decision Forest Regression, Bayesian Linear Regression, and Linear Regression. They found the Boosted Decision Tree Regression to be the best performer and are also considering adding work stress, daily physical activity, alcohol intake, and eating red meat as additional features for improving prediction. Their cohort was a Bangladesh cohort and their data was not stratified according to biological sex. In this study it was found that Huber Regressor in females and Bayesian Ridge in males were the favoured options based on the machine selection. The three differences between these two studies were the feature selections, the best machine model as well as the split between the male and female cohort.

Considering triglycerides and HDL; the research done by Palumbo et al. had favourable prediction results for triglycerides, total cholesterol, HDL and urea while having a lower accuracy for glucose and total proteins. In their methodology they used 100 k-folds of training to improve model efficiency, whereas in this study 10 k-folds was used. Their process took 23 days using two 3.6 GHz Intel ® Core™ i7–4790 CPU systems running in parallel. In terms of the ML models, like this study, Palumbo et al. also used Regression models such as Partial Least Squares Regression, and Support Vector Regression, Decision Tree, and Random Forest with the exception of the unsupervised ML Neural Networks model and the ensemble method^21^.

The final marker that did not perform optimally was UrAlbCr. The more favourable research done by Huang et al. had these features included in their model training: sex, age, BMI, duration of diabetes, smoking, alcohol, fasting plasma glucose, glycated haemoglobin, triglycerides, high-density lipoprotein cholesterol, low-density lipoprotein cholesterol, alanine aminotransferase, creatinine, systolic blood pressure, diastolic blood pressure. They found that creatinine level was the most important feature, followed by systolic and diastolic blood pressure, glycated haemoglobin, and fasting plasma glucose in predicting UrAlbCr. This study had similar features selected in predicting UrAlbCr. The markers that Huang et al. selected differently were glycated haemoglobin, low-density lipoprotein, alanine aminotransferase and diastolic blood pressure. Similarly, the research done by Huang et al. trained using 10 k-folds, using Regression Tree, Random Forest, Stochastic Gradient Boosting, and Extreme Gradient Boosting classification ML methods.

Stratifying the data by sex revealed distinct variations in variable importance. Although the reduction in predictive performance was expected due to smaller dataset sizes, the analysis provided key insights into the differential contributions of biomarkers between males and females, such as BMI, systolic blood pressure, and waist circumference. This emphasises the need for sex-specific models in medical research, as pooling data can obscure important biological nuances. These findings suggest that current diagnostic criteria for conditions like metabolic syndrome could benefit from revisiting sex-specific thresholds, potentially refining guidelines such as ATP-III, WHO, and IDF, particularly with the global rise in cardiovascular disease (CVD) and diabetes mellitus.

While sex as an input variable improved predictive ability compared to the combined data without sex as an input feature, the sex-specific models offer critical insights into how male and female physiology respond differently to risk factors. This could inform more personalised treatment strategies and refine public health approaches. By advancing the understanding of these relationships, this research supports efforts to improve diagnostic and therapeutic approaches in cardiometabolic diseases.

A limitation observed in this study was the impact of age on the data. This was particularly evident in the case of the BMI biomarker, which showed a higher prevalence of elevated BMI in the 20–29 age range compared to the 60–69 age range. To enhance the predictive capabilities of the ML model, one approach could be to stratify the data based on different age ranges which are diagnostically relevant. Another hypothesis is that this study did not stratify biomarkers according to diagnostic ranges, such as normal vs hypertensive readings for blood pressure. Training our model using specific diagnostic ranges for each biomarker may lead to more accurate predictions within those particular health conditions. The conventional approach to ML methodology typically involves the pre-selection of specific models. However, this study deviated from this by adopting a more generalised approach by selecting multiple models without bias and facilitating their competition for optimal performance confirmed by using the validation metrics. It was also noticed that features contributed towards the accuracy of a model’s prediction capability and therefore special consideration towards feature selection is a vital part of ML hyperparameter tuning. It was observed that certain model types responded well to feature selection, while others did not. The application of the RFECV methodology across all model frameworks may have led to some loss of accuracy, particularly in terms of MAPE and RMSLE as reflected in the cross-validation scores. Additionally, for model validation, 20% of the training data was reserved as a holdout set, ensuring that male test data was only evaluated on models trained with male-specific data, and similarly for female data. While we recognise the challenges associated with testing data on corresponding sex-specific models which often result in less than optimal validation score, this approach underscored the importance and value of developing distinct models for each sex.

Although a wide variety of validation metrics were used to analyse the various biomarkers, future works would include selecting a main metric with a supporting metric that suits the requirements for each biomarker. For example, for models where outliers are important in medical ranges such as blood pressure, squared metrics should feature e.g RMSE. For models with expected linear relationships such as BMI or waist circumference, metrics such as R^2^ or MAE would be a better fit. A surprising observation is that linear regression models were primarily selected as optimal models (Table 5). As shown in the above examples, non-linear regression models are a typical choice such as Random Forest. These findings could be due to the small data set. To improve validation scores and the results from the validation test, especially for underperforming models, a larger dataset (at least 10,000 entries) with greater variance should be considered in future work. Future investigations will explore two additional comparisons: one to evaluate the effect of stratifying the data by age groups on biomarker prediction, and another to assess the impact of categorising the data according to diagnostic ranges or groups. The motivation for this was inspired by the results found in the Levene, t-test and feature selection results, where it was clearly seen how data variability between the two subgroups contributed towards feature selection and therefore influenced the model prediction capabilities for the various biomarkers. An additional factor in optimising biomarker prediction is incorporating features known to improve the prediction of triglycerides, HDL, uric acid, and UrAlbCr. These features include glycated haemoglobin, C-reactive protein, presence of diabetes, hypertension, and diastolic blood pressure. After optimising the prediction models for these biomarkers, the next step would be to apply the framework outlined in this paper to predict features across different datasets. This approach would offer valuable insights into key biological markers, particularly in scenarios with limited data availability.

**Table 5:** Showing the various model categories and models used in the study

Model Category	Number of Models	Models
Linear	9	Huber Regressor, Bayesian Ridge, Ridge Regression, Linear Regression, Least Angle Regression, Orthogonal Matching Pursuit, Lasso Regression, Elastic Net, Lasso Least Angle Regression
Ensemble	6	AdaBoost Regressor, Random Forest Regressor, Extra Trees Regressor, Gradient Boosting Regressor, Light Gradient Boosting Machine, Extreme Gradient Boosting
Tree Based	2	K Neighbours Regressor, Decision Tree Regressor

This study demonstrated the application of supervised machine learning approaches in identifying sex-specific and combined-sex relationships between biomarkers. Feature analysis supported by RFECV approach revealed key interactions among biomarkers for male, female and combined groups, underscoring the importance of sex-specific models for more accurate predictions as well as to identify nuances that exist with respect to the model targets. This study also highlighted the limitations of the current feature sets in predicting triglycerides, HDL, uric acid, UrAlbCr for all 4 data set groups. These findings showed the influence of feature selection and model optimisation on prediction accuracy. Future work will include the integration of additional clinical and demographic features such as age stratification and diagnostic ranges to improve model precision. The ability to develop sex-specific predictive models paves the way for a promising avenue in the personalised medicine revolution.

## Methods

### Machine learning component

#### Data collection and preprocessing

**Fig. 11:**
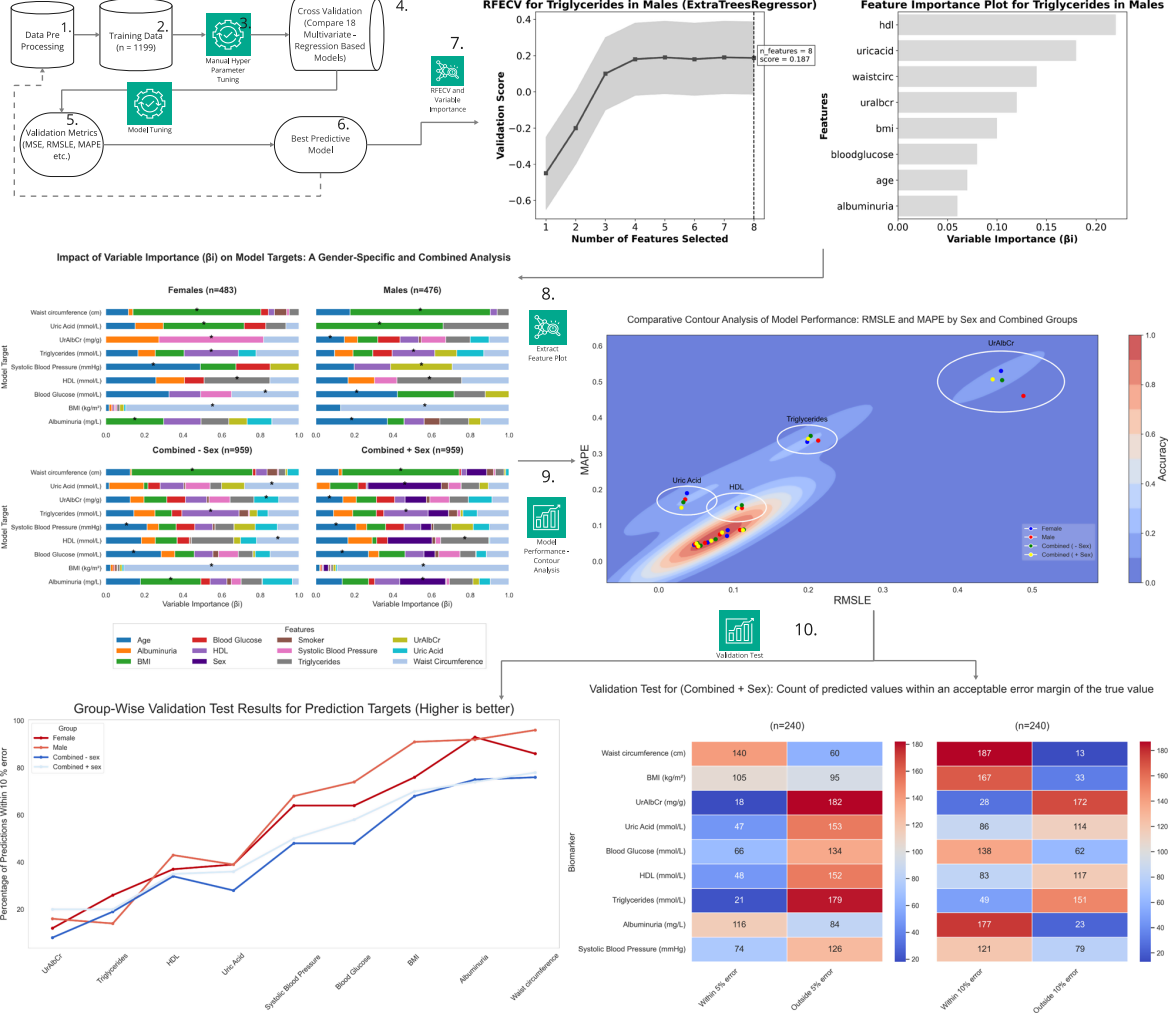
Comprehensive workflow for sex-specific predictive modelling. This illustration of the predictive modelling workflow starts with data preprocessing and hyperparameter tuning, followed by cross-validation of multivariate models. Feature selection is performed using RFECV, with model performance evaluated by MSE, RMSLE, and MAPE. Performance is further supported by sex-specific variable importance analysis, contour plots, and validation testing.

The study utilised SQL data derived from the open source National Health and Nutrition Examination Survey (NHANES), comprising 1931 participants, containing anthropometric and biomarkers associated with metabolic syndrome, type 2 diabetes (T2DM), and cardiovascular disease^22^. Preprocessing techniques were implemented to ensure data accuracy and quality. Cases with missing information were systematically eliminated to prevent potential biases. Moreover, outliers surpassing 1.5 standard deviations from the average were removed to reduce distortions in subsequent analytical processes. Data normalisation was enabled in the hyperparameter tuning during the model setup procedure and individuals aged over 80 years old were excluded due to limited sample size. Following preprocessing, the cohort comprised 1199 participants, which was then utilised for training a series of supervised regression-based ML models. The study outline is indicated in Fig. 11.

Illustration of a predictive modelling workflow, starting with data preprocessing and hyperparameter tuning, followed by cross-validation of multivariate models. Feature selection is performed using RFECV, with model performance evaluated by MSE, RMSLE, and MAPE. Performance is further supported by sex-specific variable importance analysis, contour plots, and validation testing.

#### Selection of biomarkers (Clinical and anthropometric)

For this study, we opted for a combination of anthropometric and clinical biomarkers to formulate a comprehensive predictive model applicable to both male and female subjects. In total, 14 features were initially considered for model training. These encompass five anthropometric indicators: sex, age, marital status, race, and smoking status; along with nine clinical biomarkers: blood glucose (mmol/L), HDL (mmol/L), triglycerides (mmol/L), and systolic blood pressure (mmHg), albuminuria (mg/L), UrAlbCr (mg/g), uric acid (mmol/L), waist circumference (cm), and BMI (kg/m²)

#### Model design and training procedure

Nine distinct biomarker predictive models were developed using pre-processed data from NHANES. 80% of males were used for training and 20% were reserved for testing; the same method was applied to females in the cohort. In total, 483 females were allocated to the training set and 121 to the test set, while there were 476 males in the training set and 119 in the test set.

During the data preparation phase, we constructed four distinct datasets, each corresponding to different cohorts. The first dataset combined male and female cohorts into a single dataset. The second dataset also integrated both cohorts but included sex as an input feature. To address potential sex-related disparities that could impact model performance, the combined dataset was subsequently partitioned into separate male and female cohorts. These disparities occasionally led to overfitting, where the model became overly attuned to sex-specific patterns, hindering its ability to generalise. By isolating male and female data, we sought to mitigate variations introduced by sex influences and reduce overall model error.

During the training phase, various ML models were systematically compared (Table 5) using cross-validation metrics (as illustrated in Table 6) for each of the nine biomarker targets. During the model training process, optimised models were ranked for predictive accuracy and learning rate^23^. In this method, a diverse range of models including Linear, Ensemble, and Tree-Based models were used instead of relying on predetermined ones. This comprehensive approach enabled us to effectively capture intricate biological relationships that are difficult to capture with more traditional ML approaches.

**Table 6:** Cross validation metrics used in the study

Metric	Description	Definition	Calculation
MAE	Mean Absolute Error	The mean of all absolute errors, where the absolute error is the difference between a measured value and its actual value	${\rm{MAE}}=\frac{1}{n}\mathop{\sum }\limits_{i=1}^{n}\left|{y}_{{\rm{i}}}-{ {\hat{y}} }_{{\rm{i}}}\right|$
MSE	Mean Squared Error	Calculates the average of the squares of the errors between predicted and observed values	${\rm{MSE}}=\frac{1}{n}\mathop{\sum }\limits_{i=1}^{n}{({y}_{{\rm{i}}}-{ {\hat{y}} }_{{\rm{i}}})}^{2}$
RMSE	Root Mean Squared Error	The square root of MSE, representing the square root of the average squared differences between predicted and observed values	${\rm{RMSE}}=\sqrt{\frac{1}{n}\mathop{\sum }\limits_{i=1}^{n}{({y}_{{\rm{i}}}-{ {\hat{y}} }_{{\rm{i}}})}^{2}}$
R²	R-squared	Assesses the proportion of variance in the dependent variable that is predictable from the independent variables	${{\rm{R}}}^{2}=1-\frac{\sum {({y}_{{\rm{i}}}-{ {\hat{y}} }_{{\rm{i}}})}^{2}}{\sum {({y}_{{\rm{i}}}-\bar{y})}^{2}}$
RMSLE	Root Mean Squared Log Error	The average of the squared differences between the logarithms of predicted and observed values	$\mathrm{RMSLE}=\sqrt{\frac{1}{n}{\sum }_{i=1}^{n}{(\log ({\text{y}}_{{\rm{i}}}+1)-\log ({\hat{y}}_{{\rm{i}}}+1))}^{2}}$
MAPE	Mean Absolute Percentage Error	Measures the average absolute percentage difference between predicted and observed values	${\rm{MAPE}}=\frac{100 \% }{n}\mathop{\sum }\limits_{i=1}^{n}\frac{\left|{\text{y}}_{{\rm{i}}}-{\hat{y}}_{{\rm{i}}}\right|}{{\text{y}}_{{\rm{i}}}}$

Where *n*: Total number of observations in the dataset. *y*_*i*_: Actual value of the i-th observation. ${ {\hat{y}} }_{i}$: Predicted value for the i-th observation.

Addressing data skewness during modelling involved a systematic approach integrating advanced preprocessing, feature engineering, and machine learning model evaluation techniques. By applying appropriate transformations and scaling to normalise skewed distributions and selecting algorithms less sensitive to skewness, both the accuracy and robustness of the predictive models improved. Comprehensive performance metrics and rigorous cross-validation ensured that the selected models remained resilient to the effects of skewed data. Further details on the framework can be found in the provided documentation^16–19^.

### Optimisation of biomarker prediction - Feature selection

In order to enhance the effectiveness of the nine predictive models for the chosen biomarkers, a feature selection approach called recursive feature elimination with cross-validation (RFECV) was employed alongside supervised regression-based ML.^24^. This approach removes the need for predetermined feature selection during model training. The method involves iteratively removing less important features, training the model on remaining features, and then cross-validating to assess its performance.

### Validation test on the hold-out set

A validation test was conducted on a 20% hold-out set to evaluate whether the predicted values fell within a ± 5% or ±10% error margin of the actual values. The results were visualised using a heatmap, showing the number of individuals from the hold-out sets—119 males and 121 females—derived from sex-specific data sets who fell within these error margins. This method was also applied to the combined dataset, which included a total of 240 individuals (both male and female). The percentage of individuals within each error margin was then calculated, and predictions were considered acceptable if more than 60% of individuals fell within the ±10% error margin in the initial assessment.

## Supplementary information

Supplementary Information

## Data Availability

All the data used in this study can be found in the NHANES database (https://data.world/rhoyt/librehealth-educational-ehr), which is an open source database and freely available.

## Code availability

The code is protected by intellectual property rights. However, we have shared the code framework and methods used in order to assist in experimental analysis if required in our github repository https://github.com/SapioSentient/Biomarker-Machine-Learning-Data.git. and references^16–19^.
