# Supplementary material for: A supervised machine learning approach with feature selection for sex-specific biomarker prediction
Source: NPJ Syst Biol Appl. 2025 Jul 1;11:69. doi: 10.1038/s41540-025-00523-z (PMC12219308; doi:10.1038/s41540-025-00523-z)
Supplement: Supplementary file 1 — Supplementary Information [file 41540_2025_523_MOESM1_ESM.docx]

**SUPPLEMENTARY: A Supervised Machine Learning Approach with Feature Selection for Sex-Specific Biomarker Prediction**

**Supplementary Table 1.** Cross-validation results of all models tested, including performance metrics grouped by biomarker and sex.

| Gender of training data | Optimal Model | Biomarker | Model_ID | Model | MAE | MSE | RMSE | R2 | RMSLE | MAPE | TT (Sec) |
| --- | --- | --- | --- | --- | --- | --- | --- | --- | --- | --- | --- |
| female | TRUE | waistcirc | br | Bayesian Ridge | 4.4996 | 33.6396 | 5.7615 | 0.8285 | 0.0587 | 0.0478 | 0.007 |
| female | FALSE | waistcirc | ridge | Ridge Regression | 4.5002 | 33.6639 | 5.7632 | 0.8283 | 0.0587 | 0.0478 | 0.006 |
| female | FALSE | waistcirc | lr | Linear Regression | 4.5014 | 33.7078 | 5.7664 | 0.8281 | 0.0587 | 0.0478 | 0.339 |
| female | FALSE | waistcirc | lar | Least Angle Regression | 4.5014 | 33.7078 | 5.7664 | 0.8281 | 0.0587 | 0.0478 | 0.007 |
| female | FALSE | waistcirc | ada | AdaBoost Regressor | 4.5683 | 32.767 | 5.7029 | 0.8272 | 0.0586 | 0.0485 | 0.014 |
| female | FALSE | waistcirc | huber | Huber Regressor | 4.5083 | 34.0597 | 5.7941 | 0.8267 | 0.0589 | 0.0479 | 0.007 |
| female | FALSE | waistcirc | et | Extra Trees Regressor | 4.5675 | 33.7961 | 5.773 | 0.819 | 0.0586 | 0.0484 | 0.031 |
| female | FALSE | waistcirc | rf | Random Forest Regressor | 4.5594 | 34.1456 | 5.8095 | 0.8177 | 0.0592 | 0.0483 | 0.045 |
| female | FALSE | waistcirc | gbr | Gradient Boosting Regressor | 4.5752 | 34.9842 | 5.89 | 0.8157 | 0.06 | 0.0484 | 0.016 |
| female | FALSE | waistcirc | lightgbm | Light Gradient Boosting Machine | 4.9069 | 38.977 | 6.1962 | 0.7924 | 0.0638 | 0.0521 | 0.043 |
| female | FALSE | waistcirc | omp | Orthogonal Matching Pursuit | 4.973 | 40.3199 | 6.3065 | 0.7903 | 0.0647 | 0.0529 | 0.006 |
| female | FALSE | waistcirc | xgboost | Extreme Gradient Boosting | 4.921 | 40.22 | 6.2997 | 0.787 | 0.0643 | 0.0522 | 0.023 |
| female | FALSE | waistcirc | knn | K Neighbors Regressor | 5.7566 | 54.5701 | 7.3587 | 0.7198 | 0.0768 | 0.0618 | 0.009 |
| female | FALSE | waistcirc | dt | Decision Tree Regressor | 6.3403 | 63.9581 | 7.9631 | 0.6656 | 0.082 | 0.0675 | 0.017 |
| female | FALSE | waistcirc | par | Passive Aggressive Regressor | 8.7912 | 155.4287 | 11.5109 | 0.239 | 0.1147 | 0.0938 | 0.006 |
| female | FALSE | waistcirc | lasso | Lasso Regression | 11.4493 | 202.0238 | 14.1752 | -0.02 | 0.1485 | 0.1234 | 0.155 |
| female | FALSE | waistcirc | en | Elastic Net | 11.4493 | 202.0238 | 14.1752 | -0.02 | 0.1485 | 0.1234 | 0.006 |
| female | FALSE | waistcirc | llar | Lasso Least Angle Regression | 11.4493 | 202.0238 | 14.1752 | -0.02 | 0.1485 | 0.1234 | 0.007 |
| female | FALSE | waistcirc | dummy | Dummy Regressor | 11.4493 | 202.0238 | 14.1752 | -0.02 | 0.1485 | 0.1234 | 0.006 |
| female | TRUE | bmi | gbr | Gradient Boosting Regressor | 1.9051 | 6.2426 | 2.4796 | 0.8094 | 0.0821 | 0.0674 | 0.018 |
| female | FALSE | bmi | et | Extra Trees Regressor | 1.9479 | 6.5097 | 2.5354 | 0.8033 | 0.0828 | 0.0684 | 0.031 |
| female | FALSE | bmi | ada | AdaBoost Regressor | 1.9656 | 6.7001 | 2.5698 | 0.7955 | 0.0841 | 0.0693 | 0.014 |
| female | FALSE | bmi | rf | Random Forest Regressor | 1.9983 | 6.7671 | 2.5834 | 0.793 | 0.0845 | 0.0703 | 0.046 |
| female | FALSE | bmi | br | Bayesian Ridge | 1.9449 | 6.8254 | 2.5805 | 0.7859 | 0.0823 | 0.0676 | 0.007 |
| female | FALSE | bmi | ridge | Ridge Regression | 1.946 | 6.8424 | 2.5835 | 0.7853 | 0.0823 | 0.0677 | 0.006 |
| female | FALSE | bmi | lr | Linear Regression | 1.9473 | 6.8631 | 2.5872 | 0.7846 | 0.0823 | 0.0677 | 0.338 |
| female | FALSE | bmi | lar | Least Angle Regression | 1.9473 | 6.8631 | 2.5872 | 0.7846 | 0.0823 | 0.0677 | 0.006 |
| female | FALSE | bmi | lightgbm | Light Gradient Boosting Machine | 2.0545 | 7.1107 | 2.6517 | 0.7828 | 0.0862 | 0.0719 | 0.045 |
| female | FALSE | bmi | huber | Huber Regressor | 1.9561 | 6.9343 | 2.5999 | 0.7827 | 0.0827 | 0.0678 | 0.007 |
| female | FALSE | bmi | omp | Orthogonal Matching Pursuit | 1.9877 | 7.1024 | 2.64 | 0.7784 | 0.085 | 0.0694 | 0.007 |
| female | FALSE | bmi | xgboost | Extreme Gradient Boosting | 2.1166 | 8.0114 | 2.8009 | 0.7574 | 0.0915 | 0.0746 | 0.025 |
| female | FALSE | bmi | knn | K Neighbors Regressor | 2.5979 | 11.41 | 3.3548 | 0.663 | 0.112 | 0.0923 | 0.009 |
| female | FALSE | bmi | dt | Decision Tree Regressor | 2.6647 | 12.171 | 3.4664 | 0.6172 | 0.1141 | 0.0951 | 0.009 |
| female | FALSE | bmi | par | Passive Aggressive Regressor | 3.5217 | 25.6678 | 4.837 | 0.1915 | 0.1454 | 0.1237 | 0.007 |
| female | FALSE | bmi | lasso | Lasso Regression | 4.7779 | 34.9059 | 5.878 | -0.0237 | 0.1999 | 0.1729 | 0.157 |
| female | FALSE | bmi | en | Elastic Net | 4.7779 | 34.9059 | 5.878 | -0.0237 | 0.1999 | 0.1729 | 0.007 |
| female | FALSE | bmi | llar | Lasso Least Angle Regression | 4.7779 | 34.9059 | 5.878 | -0.0237 | 0.1999 | 0.1729 | 0.006 |
| female | FALSE | bmi | dummy | Dummy Regressor | 4.7779 | 34.9059 | 5.878 | -0.0237 | 0.1999 | 0.1729 | 0.006 |
| female | TRUE | uralbcr | lr | Linear Regression | 3.3678 | 22.1941 | 4.6856 | -0.0504 | 0.4558 | 0.4492 | 0.31 |
| female | FALSE | uralbcr | lar | Least Angle Regression | 3.3678 | 22.1941 | 4.6856 | -0.0504 | 0.4558 | 0.4492 | 0.008 |
| female | FALSE | uralbcr | ridge | Ridge Regression | 3.3678 | 22.1945 | 4.6857 | -0.0505 | 0.4558 | 0.4492 | 0.006 |
| female | FALSE | uralbcr | huber | Huber Regressor | 3.3698 | 22.1958 | 4.6857 | -0.0508 | 0.4558 | 0.4477 | 0.006 |
| female | FALSE | uralbcr | br | Bayesian Ridge | 3.3697 | 22.2538 | 4.6919 | -0.0529 | 0.4557 | 0.4491 | 0.006 |
| female | FALSE | uralbcr | ada | AdaBoost Regressor | 3.3675 | 22.3369 | 4.7048 | -0.0601 | 0.4567 | 0.4495 | 0.014 |
| female | FALSE | uralbcr | omp | Orthogonal Matching Pursuit | 3.3855 | 22.4094 | 4.7094 | -0.061 | 0.4575 | 0.4509 | 0.006 |
| female | FALSE | uralbcr | lasso | Lasso Regression | 3.4327 | 22.9222 | 4.7624 | -0.0838 | 0.4623 | 0.4578 | 0.154 |
| female | FALSE | uralbcr | llar | Lasso Least Angle Regression | 3.4327 | 22.9222 | 4.7624 | -0.0838 | 0.4623 | 0.4578 | 0.006 |
| female | FALSE | uralbcr | en | Elastic Net | 3.4327 | 22.9222 | 4.7624 | -0.0838 | 0.4623 | 0.4578 | 0.006 |
| female | FALSE | uralbcr | dummy | Dummy Regressor | 3.4327 | 22.9222 | 4.7624 | -0.0838 | 0.4623 | 0.4578 | 0.005 |
| female | FALSE | uralbcr | gbr | Gradient Boosting Regressor | 3.632 | 24.2297 | 4.9041 | -0.1589 | 0.4838 | 0.4923 | 0.011 |
| female | FALSE | uralbcr | rf | Random Forest Regressor | 3.6286 | 24.5388 | 4.9362 | -0.1744 | 0.49 | 0.4937 | 0.033 |
| female | FALSE | uralbcr | lightgbm | Light Gradient Boosting Machine | 3.6405 | 24.9742 | 4.9742 | -0.1928 | 0.497 | 0.4928 | 0.031 |
| female | FALSE | uralbcr | knn | K Neighbors Regressor | 3.6749 | 25.2686 | 5.0055 | -0.2136 | 0.498 | 0.492 | 0.008 |
| female | FALSE | uralbcr | et | Extra Trees Regressor | 3.8177 | 26.3287 | 5.1118 | -0.2613 | 0.5104 | 0.5216 | 0.027 |
| female | FALSE | uralbcr | xgboost | Extreme Gradient Boosting | 4.2168 | 32.6205 | 5.6935 | -0.5655 | 0.5603 | 0.5835 | 0.021 |
| female | FALSE | uralbcr | dt | Decision Tree Regressor | 4.9693 | 41.0175 | 6.3814 | -0.9657 | 0.6379 | 0.7057 | 0.006 |
| female | FALSE | uralbcr | par | Passive Aggressive Regressor | 312.4051 | 34947798.56 | 1877.4977 | -1521651.244 | 0.9128 | 40.2465 | 0.006 |
| female | TRUE | uricacid | huber | Huber Regressor | 0.0284 | 0.0014 | 0.0367 | 0.1253 | 0.0312 | 0.1795 | 0.007 |
| female | FALSE | uricacid | br | Bayesian Ridge | 0.0285 | 0.0014 | 0.0367 | 0.1243 | 0.0312 | 0.1795 | 0.006 |
| female | FALSE | uricacid | ridge | Ridge Regression | 0.0285 | 0.0014 | 0.0367 | 0.1232 | 0.0312 | 0.1795 | 0.008 |
| female | FALSE | uricacid | lr | Linear Regression | 0.0285 | 0.0014 | 0.0367 | 0.1231 | 0.0312 | 0.1795 | 0.389 |
| female | FALSE | uricacid | ada | AdaBoost Regressor | 0.0289 | 0.0014 | 0.0369 | 0.123 | 0.0313 | 0.1797 | 0.013 |
| female | FALSE | uricacid | lar | Least Angle Regression | 0.0292 | 0.0015 | 0.0379 | 0.0715 | 0.0322 | 0.1832 | 0.006 |
| female | FALSE | uricacid | omp | Orthogonal Matching Pursuit | 0.0292 | 0.0015 | 0.0381 | 0.0673 | 0.0323 | 0.1841 | 0.006 |
| female | FALSE | uricacid | rf | Random Forest Regressor | 0.0294 | 0.0015 | 0.0379 | 0.0662 | 0.0322 | 0.185 | 0.043 |
| female | FALSE | uricacid | et | Extra Trees Regressor | 0.0299 | 0.0015 | 0.0381 | 0.0547 | 0.0324 | 0.1885 | 0.027 |
| female | FALSE | uricacid | gbr | Gradient Boosting Regressor | 0.0301 | 0.0015 | 0.0382 | 0.0455 | 0.0325 | 0.1891 | 0.014 |
| female | FALSE | uricacid | lightgbm | Light Gradient Boosting Machine | 0.0308 | 0.0015 | 0.039 | 0.0021 | 0.0331 | 0.1928 | 0.035 |
| female | FALSE | uricacid | lasso | Lasso Regression | 0.0308 | 0.0016 | 0.0402 | -0.0326 | 0.0341 | 0.194 | 0.156 |
| female | FALSE | uricacid | en | Elastic Net | 0.0308 | 0.0016 | 0.0402 | -0.0326 | 0.0341 | 0.194 | 0.006 |
| female | FALSE | uricacid | llar | Lasso Least Angle Regression | 0.0308 | 0.0016 | 0.0402 | -0.0326 | 0.0341 | 0.194 | 0.006 |
| female | FALSE | uricacid | dummy | Dummy Regressor | 0.0308 | 0.0016 | 0.0402 | -0.0326 | 0.0341 | 0.194 | 0.005 |
| female | FALSE | uricacid | knn | K Neighbors Regressor | 0.0307 | 0.0016 | 0.0396 | -0.0344 | 0.0337 | 0.1941 | 0.008 |
| female | FALSE | uricacid | xgboost | Extreme Gradient Boosting | 0.0323 | 0.0017 | 0.0414 | -0.1342 | 0.0352 | 0.2023 | 0.023 |
| female | FALSE | uricacid | dt | Decision Tree Regressor | 0.0422 | 0.003 | 0.0541 | -1.0127 | 0.0458 | 0.2646 | 0.006 |
| female | FALSE | uricacid | par | Passive Aggressive Regressor | 0.1288 | 0.018 | 0.1339 | -10.864 | 0.1207 | 0.7739 | 0.006 |
| female | TRUE | bloodglucose | lr | Linear Regression | 0.3969 | 0.2678 | 0.5146 | 0.265 | 0.0792 | 0.0737 | 0.437 |
| female | FALSE | bloodglucose | ridge | Ridge Regression | 0.3969 | 0.2678 | 0.5146 | 0.265 | 0.0792 | 0.0736 | 0.006 |
| female | FALSE | bloodglucose | lar | Least Angle Regression | 0.3969 | 0.2678 | 0.5146 | 0.265 | 0.0792 | 0.0737 | 0.006 |
| female | FALSE | bloodglucose | huber | Huber Regressor | 0.3967 | 0.2678 | 0.5148 | 0.265 | 0.0793 | 0.0738 | 0.006 |
| female | FALSE | bloodglucose | br | Bayesian Ridge | 0.3967 | 0.2679 | 0.5149 | 0.2646 | 0.0793 | 0.0736 | 0.006 |
| female | FALSE | bloodglucose | ada | AdaBoost Regressor | 0.421 | 0.3003 | 0.5448 | 0.175 | 0.0839 | 0.0776 | 0.013 |
| female | FALSE | bloodglucose | rf | Random Forest Regressor | 0.4222 | 0.3012 | 0.5462 | 0.171 | 0.0842 | 0.0782 | 0.037 |
| female | FALSE | bloodglucose | omp | Orthogonal Matching Pursuit | 0.4172 | 0.3042 | 0.5499 | 0.1648 | 0.0844 | 0.0772 | 0.005 |
| female | FALSE | bloodglucose | et | Extra Trees Regressor | 0.4274 | 0.3068 | 0.552 | 0.1555 | 0.085 | 0.0792 | 0.025 |
| female | FALSE | bloodglucose | gbr | Gradient Boosting Regressor | 0.4264 | 0.3068 | 0.5517 | 0.1526 | 0.0851 | 0.0792 | 0.013 |
| female | FALSE | bloodglucose | knn | K Neighbors Regressor | 0.4313 | 0.3123 | 0.5565 | 0.1424 | 0.086 | 0.08 | 0.008 |
| female | FALSE | bloodglucose | lightgbm | Light Gradient Boosting Machine | 0.4457 | 0.3291 | 0.5707 | 0.0919 | 0.0881 | 0.0827 | 0.035 |
| female | FALSE | bloodglucose | xgboost | Extreme Gradient Boosting | 0.4632 | 0.3469 | 0.5862 | 0.0463 | 0.0908 | 0.0861 | 0.022 |
| female | FALSE | bloodglucose | lasso | Lasso Regression | 0.4618 | 0.3723 | 0.6086 | -0.0194 | 0.0936 | 0.0853 | 0.149 |
| female | FALSE | bloodglucose | en | Elastic Net | 0.4618 | 0.3723 | 0.6086 | -0.0194 | 0.0936 | 0.0853 | 0.006 |
| female | FALSE | bloodglucose | llar | Lasso Least Angle Regression | 0.4618 | 0.3723 | 0.6086 | -0.0194 | 0.0936 | 0.0853 | 0.006 |
| female | FALSE | bloodglucose | dummy | Dummy Regressor | 0.4618 | 0.3723 | 0.6086 | -0.0194 | 0.0936 | 0.0853 | 0.006 |
| female | FALSE | bloodglucose | dt | Decision Tree Regressor | 0.5782 | 0.5628 | 0.7465 | -0.5567 | 0.1156 | 0.1082 | 0.006 |
| female | FALSE | bloodglucose | par | Passive Aggressive Regressor | 1.1937 | 18.5711 | 2.3543 | -51.6504 | 0.1992 | 0.2158 | 0.007 |
| female | TRUE | hdl | br | Bayesian Ridge | 0.2245 | 0.0776 | 0.2781 | 0.2157 | 0.1108 | 0.1556 | 0.007 |
| female | FALSE | hdl | ridge | Ridge Regression | 0.2244 | 0.0776 | 0.278 | 0.2153 | 0.1108 | 0.1555 | 0.006 |
| female | FALSE | hdl | lr | Linear Regression | 0.2244 | 0.0776 | 0.278 | 0.2152 | 0.1108 | 0.1555 | 0.331 |
| female | FALSE | hdl | lar | Least Angle Regression | 0.2244 | 0.0776 | 0.278 | 0.2152 | 0.1108 | 0.1555 | 0.006 |
| female | FALSE | hdl | huber | Huber Regressor | 0.2242 | 0.0779 | 0.2785 | 0.2101 | 0.111 | 0.1549 | 0.006 |
| female | FALSE | hdl | ada | AdaBoost Regressor | 0.2316 | 0.0817 | 0.2854 | 0.1791 | 0.1143 | 0.163 | 0.012 |
| female | FALSE | hdl | et | Extra Trees Regressor | 0.2319 | 0.082 | 0.2859 | 0.1739 | 0.1141 | 0.1612 | 0.027 |
| female | FALSE | hdl | rf | Random Forest Regressor | 0.2313 | 0.0827 | 0.287 | 0.1654 | 0.1145 | 0.1607 | 0.04 |
| female | FALSE | hdl | knn | K Neighbors Regressor | 0.2359 | 0.0849 | 0.2907 | 0.1461 | 0.1159 | 0.1641 | 0.008 |
| female | FALSE | hdl | gbr | Gradient Boosting Regressor | 0.2372 | 0.0854 | 0.2916 | 0.1348 | 0.1159 | 0.1642 | 0.014 |
| female | FALSE | hdl | lightgbm | Light Gradient Boosting Machine | 0.2407 | 0.0877 | 0.2954 | 0.1141 | 0.1176 | 0.1661 | 0.04 |
| female | FALSE | hdl | omp | Orthogonal Matching Pursuit | 0.2465 | 0.0898 | 0.2991 | 0.1032 | 0.1193 | 0.1722 | 0.006 |
| female | FALSE | hdl | xgboost | Extreme Gradient Boosting | 0.2459 | 0.0959 | 0.3086 | 0.0385 | 0.123 | 0.1697 | 0.025 |
| female | FALSE | hdl | lasso | Lasso Regression | 0.2654 | 0.1046 | 0.3227 | -0.035 | 0.1289 | 0.1863 | 0.156 |
| female | FALSE | hdl | en | Elastic Net | 0.2654 | 0.1046 | 0.3227 | -0.035 | 0.1289 | 0.1863 | 0.006 |
| female | FALSE | hdl | llar | Lasso Least Angle Regression | 0.2654 | 0.1046 | 0.3227 | -0.035 | 0.1289 | 0.1863 | 0.006 |
| female | FALSE | hdl | dummy | Dummy Regressor | 0.2654 | 0.1046 | 0.3227 | -0.035 | 0.1289 | 0.1863 | 0.006 |
| female | FALSE | hdl | dt | Decision Tree Regressor | 0.3135 | 0.1535 | 0.3912 | -0.5344 | 0.1554 | 0.2172 | 0.006 |
| female | FALSE | hdl | par | Passive Aggressive Regressor | 0.304 | 0.1674 | 0.3867 | -0.7483 | 0.1511 | 0.2161 | 0.006 |
| female | TRUE | triglycerides | lr | Linear Regression | 0.3261 | 0.1831 | 0.4259 | 0.2365 | 0.1896 | 0.3211 | 0.37 |
| female | FALSE | triglycerides | lar | Least Angle Regression | 0.3261 | 0.1831 | 0.4259 | 0.2365 | 0.1896 | 0.3211 | 0.006 |
| female | FALSE | triglycerides | ridge | Ridge Regression | 0.3261 | 0.1831 | 0.426 | 0.2364 | 0.1896 | 0.3211 | 0.006 |
| female | FALSE | triglycerides | huber | Huber Regressor | 0.3249 | 0.1829 | 0.4256 | 0.236 | 0.1899 | 0.318 | 0.006 |
| female | FALSE | triglycerides | br | Bayesian Ridge | 0.3265 | 0.184 | 0.427 | 0.2333 | 0.1898 | 0.3216 | 0.006 |
| female | FALSE | triglycerides | ada | AdaBoost Regressor | 0.3377 | 0.1927 | 0.4367 | 0.1982 | 0.1933 | 0.332 | 0.017 |
| female | FALSE | triglycerides | rf | Random Forest Regressor | 0.3434 | 0.1972 | 0.4423 | 0.1739 | 0.1972 | 0.3392 | 0.042 |
| female | FALSE | triglycerides | et | Extra Trees Regressor | 0.3473 | 0.1991 | 0.4448 | 0.1612 | 0.1985 | 0.3436 | 0.028 |
| female | FALSE | triglycerides | gbr | Gradient Boosting Regressor | 0.3478 | 0.2022 | 0.4474 | 0.1551 | 0.1996 | 0.3438 | 0.015 |
| female | FALSE | triglycerides | lightgbm | Light Gradient Boosting Machine | 0.3628 | 0.214 | 0.4605 | 0.0999 | 0.2062 | 0.3581 | 0.033 |
| female | FALSE | triglycerides | omp | Orthogonal Matching Pursuit | 0.3618 | 0.221 | 0.4683 | 0.0807 | 0.2078 | 0.3622 | 0.006 |
| female | FALSE | triglycerides | knn | K Neighbors Regressor | 0.3589 | 0.219 | 0.4664 | 0.0798 | 0.2078 | 0.3485 | 0.008 |
| female | FALSE | triglycerides | xgboost | Extreme Gradient Boosting | 0.3686 | 0.2329 | 0.4817 | 0.0084 | 0.2155 | 0.3675 | 0.026 |
| female | FALSE | triglycerides | lasso | Lasso Regression | 0.3933 | 0.2588 | 0.5063 | -0.0665 | 0.2248 | 0.3969 | 0.155 |
| female | FALSE | triglycerides | en | Elastic Net | 0.3933 | 0.2588 | 0.5063 | -0.0665 | 0.2248 | 0.3969 | 0.006 |
| female | FALSE | triglycerides | llar | Lasso Least Angle Regression | 0.3933 | 0.2588 | 0.5063 | -0.0665 | 0.2248 | 0.3969 | 0.006 |
| female | FALSE | triglycerides | dummy | Dummy Regressor | 0.3933 | 0.2588 | 0.5063 | -0.0665 | 0.2248 | 0.3969 | 0.006 |
| female | FALSE | triglycerides | dt | Decision Tree Regressor | 0.4561 | 0.3353 | 0.576 | -0.442 | 0.261 | 0.4737 | 0.006 |
| female | FALSE | triglycerides | par | Passive Aggressive Regressor | 0.5044 | 0.4282 | 0.625 | -0.9323 | 0.2878 | 0.4991 | 0.006 |
| female | TRUE | albuminuria | br | Bayesian Ridge | 0.2097 | 0.068 | 0.2589 | 0.1208 | 0.0498 | 0.0501 | 0.006 |
| female | FALSE | albuminuria | ridge | Ridge Regression | 0.2102 | 0.0682 | 0.2592 | 0.1184 | 0.0498 | 0.0502 | 0.006 |
| female | FALSE | albuminuria | lr | Linear Regression | 0.2102 | 0.0682 | 0.2592 | 0.1182 | 0.0498 | 0.0502 | 0.354 |
| female | FALSE | albuminuria | lar | Least Angle Regression | 0.2102 | 0.0682 | 0.2592 | 0.1182 | 0.0498 | 0.0502 | 0.006 |
| female | FALSE | albuminuria | huber | Huber Regressor | 0.2101 | 0.0689 | 0.2603 | 0.1133 | 0.0501 | 0.0502 | 0.006 |
| female | FALSE | albuminuria | omp | Orthogonal Matching Pursuit | 0.213 | 0.0709 | 0.2641 | 0.088 | 0.0508 | 0.051 | 0.006 |
| female | FALSE | albuminuria | rf | Random Forest Regressor | 0.2178 | 0.0754 | 0.2722 | 0.0236 | 0.0523 | 0.052 | 0.041 |
| female | FALSE | albuminuria | ada | AdaBoost Regressor | 0.222 | 0.0768 | 0.2748 | -0.0006 | 0.0528 | 0.0531 | 0.013 |
| female | FALSE | albuminuria | gbr | Gradient Boosting Regressor | 0.2205 | 0.0774 | 0.2762 | -0.0057 | 0.053 | 0.0526 | 0.016 |
| female | FALSE | albuminuria | et | Extra Trees Regressor | 0.22 | 0.078 | 0.2768 | -0.0103 | 0.0532 | 0.0526 | 0.027 |
| female | FALSE | albuminuria | lasso | Lasso Regression | 0.2244 | 0.081 | 0.2825 | -0.045 | 0.0543 | 0.0537 | 0.153 |
| female | FALSE | albuminuria | en | Elastic Net | 0.2244 | 0.081 | 0.2825 | -0.045 | 0.0543 | 0.0537 | 0.006 |
| female | FALSE | albuminuria | llar | Lasso Least Angle Regression | 0.2244 | 0.081 | 0.2825 | -0.045 | 0.0543 | 0.0537 | 0.006 |
| female | FALSE | albuminuria | dummy | Dummy Regressor | 0.2244 | 0.081 | 0.2825 | -0.045 | 0.0543 | 0.0537 | 0.006 |
| female | FALSE | albuminuria | knn | K Neighbors Regressor | 0.2282 | 0.0818 | 0.2841 | -0.062 | 0.0546 | 0.0546 | 0.008 |
| female | FALSE | albuminuria | lightgbm | Light Gradient Boosting Machine | 0.2358 | 0.0879 | 0.2944 | -0.1442 | 0.0566 | 0.0563 | 0.032 |
| female | FALSE | albuminuria | xgboost | Extreme Gradient Boosting | 0.2326 | 0.0874 | 0.2944 | -0.1525 | 0.0564 | 0.0553 | 0.029 |
| female | FALSE | albuminuria | dt | Decision Tree Regressor | 0.29 | 0.1345 | 0.3647 | -0.7788 | 0.0699 | 0.069 | 0.006 |
| female | FALSE | albuminuria | par | Passive Aggressive Regressor | 0.3639 | 0.2268 | 0.4524 | -2.0104 | 0.0884 | 0.086 | 0.006 |
| female | TRUE | sys_bp | huber | Huber Regressor | 10.0828 | 170.2879 | 12.9701 | 0.2344 | 0.1074 | 0.0857 | 0.006 |
| female | FALSE | sys_bp | lr | Linear Regression | 10.0638 | 170.9402 | 12.9904 | 0.232 | 0.1075 | 0.0853 | 0.344 |
| female | FALSE | sys_bp | lar | Least Angle Regression | 10.0638 | 170.9402 | 12.9904 | 0.232 | 0.1075 | 0.0853 | 0.006 |
| female | FALSE | sys_bp | ridge | Ridge Regression | 10.0639 | 170.9557 | 12.991 | 0.2319 | 0.1075 | 0.0853 | 0.005 |
| female | FALSE | sys_bp | br | Bayesian Ridge | 10.0639 | 170.9493 | 12.9907 | 0.2319 | 0.1075 | 0.0853 | 0.006 |
| female | FALSE | sys_bp | omp | Orthogonal Matching Pursuit | 10.3149 | 183.1064 | 13.4385 | 0.1793 | 0.111 | 0.0874 | 0.005 |
| female | FALSE | sys_bp | ada | AdaBoost Regressor | 10.474 | 185.8663 | 13.5292 | 0.1652 | 0.1119 | 0.0885 | 0.012 |
| female | FALSE | sys_bp | gbr | Gradient Boosting Regressor | 10.6503 | 193.6429 | 13.8297 | 0.1295 | 0.1147 | 0.0906 | 0.014 |
| female | FALSE | sys_bp | xgboost | Extreme Gradient Boosting | 10.6246 | 195.0682 | 13.8709 | 0.1247 | 0.1144 | 0.0897 | 0.019 |
| female | FALSE | sys_bp | rf | Random Forest Regressor | 10.7112 | 199.0046 | 14.0173 | 0.1043 | 0.1162 | 0.0908 | 0.039 |
| female | FALSE | sys_bp | knn | K Neighbors Regressor | 11.1059 | 205.4259 | 14.2177 | 0.0763 | 0.1183 | 0.0941 | 0.008 |
| female | FALSE | sys_bp | lightgbm | Light Gradient Boosting Machine | 10.9872 | 208.2781 | 14.331 | 0.0634 | 0.1192 | 0.0936 | 0.048 |
| female | FALSE | sys_bp | et | Extra Trees Regressor | 11.1285 | 212.8959 | 14.4961 | 0.0422 | 0.1204 | 0.0945 | 0.026 |
| female | FALSE | sys_bp | lasso | Lasso Regression | 11.6923 | 233.8831 | 15.1932 | -0.0462 | 0.1252 | 0.0984 | 0.15 |
| female | FALSE | sys_bp | en | Elastic Net | 11.6923 | 233.8831 | 15.1932 | -0.0462 | 0.1252 | 0.0984 | 0.006 |
| female | FALSE | sys_bp | llar | Lasso Least Angle Regression | 11.6923 | 233.8831 | 15.1932 | -0.0462 | 0.1252 | 0.0984 | 0.007 |
| female | FALSE | sys_bp | dummy | Dummy Regressor | 11.6923 | 233.8831 | 15.1932 | -0.0462 | 0.1252 | 0.0984 | 0.005 |
| female | FALSE | sys_bp | dt | Decision Tree Regressor | 14.3949 | 347.2034 | 18.5357 | -0.574 | 0.1531 | 0.1231 | 0.006 |
| male | TRUE | waistcirc | huber | Huber Regressor | 3.9131 | 23.966 | 4.8551 | 0.8643 | 0.0491 | 0.0404 | 0.006 |
| male | FALSE | waistcirc | ridge | Ridge Regression | 3.9059 | 24.0116 | 4.8587 | 0.864 | 0.0492 | 0.0403 | 0.006 |
| male | FALSE | waistcirc | br | Bayesian Ridge | 3.9061 | 24.0145 | 4.8591 | 0.864 | 0.0492 | 0.0403 | 0.006 |
| male | FALSE | waistcirc | lr | Linear Regression | 3.907 | 24.0288 | 4.8607 | 0.8639 | 0.0492 | 0.0403 | 0.333 |
| male | FALSE | waistcirc | lar | Least Angle Regression | 3.907 | 24.0288 | 4.8607 | 0.8639 | 0.0492 | 0.0403 | 0.006 |
| male | FALSE | waistcirc | gbr | Gradient Boosting Regressor | 4.1211 | 26.5129 | 5.1081 | 0.8515 | 0.0521 | 0.0426 | 0.013 |
| male | FALSE | waistcirc | ada | AdaBoost Regressor | 4.1383 | 27.372 | 5.1882 | 0.847 | 0.0533 | 0.043 | 0.012 |
| male | FALSE | waistcirc | et | Extra Trees Regressor | 4.192 | 27.77 | 5.2292 | 0.8445 | 0.0532 | 0.0433 | 0.026 |
| male | FALSE | waistcirc | rf | Random Forest Regressor | 4.2254 | 28.2354 | 5.2645 | 0.8417 | 0.0536 | 0.0437 | 0.034 |
| male | FALSE | waistcirc | lightgbm | Light Gradient Boosting Machine | 4.3269 | 29.3892 | 5.3667 | 0.8357 | 0.0544 | 0.0447 | 0.042 |
| male | FALSE | waistcirc | xgboost | Extreme Gradient Boosting | 4.4434 | 31.0899 | 5.5301 | 0.826 | 0.0562 | 0.0459 | 0.022 |
| male | FALSE | waistcirc | knn | K Neighbors Regressor | 4.3858 | 31.6675 | 5.5647 | 0.8225 | 0.0563 | 0.0453 | 0.008 |
| male | FALSE | waistcirc | omp | Orthogonal Matching Pursuit | 4.5201 | 32.7414 | 5.6866 | 0.8182 | 0.0582 | 0.047 | 0.006 |
| male | FALSE | waistcirc | par | Passive Aggressive Regressor | 4.8847 | 41.6714 | 6.1185 | 0.7624 | 0.0615 | 0.0502 | 0.006 |
| male | FALSE | waistcirc | dt | Decision Tree Regressor | 5.9997 | 57.2527 | 7.5442 | 0.6761 | 0.0766 | 0.0621 | 0.006 |
| male | FALSE | waistcirc | lasso | Lasso Regression | 10.9681 | 188.7326 | 13.6909 | -0.0279 | 0.1387 | 0.1141 | 0.143 |
| male | FALSE | waistcirc | en | Elastic Net | 10.9681 | 188.7326 | 13.6909 | -0.0279 | 0.1387 | 0.1141 | 0.006 |
| male | FALSE | waistcirc | llar | Lasso Least Angle Regression | 10.9681 | 188.7326 | 13.6909 | -0.0279 | 0.1387 | 0.1141 | 0.005 |
| male | FALSE | waistcirc | dummy | Dummy Regressor | 10.9681 | 188.7326 | 13.6909 | -0.0279 | 0.1387 | 0.1141 | 0.006 |
| male | TRUE | bmi | ridge | Ridge Regression | 1.4964 | 3.5514 | 1.8728 | 0.8376 | 0.0657 | 0.0551 | 0.005 |
| male | FALSE | bmi | br | Bayesian Ridge | 1.4966 | 3.556 | 1.8737 | 0.8374 | 0.0657 | 0.0551 | 0.006 |
| male | FALSE | bmi | lr | Linear Regression | 1.4967 | 3.558 | 1.8742 | 0.8373 | 0.0657 | 0.055 | 0.316 |
| male | FALSE | bmi | lar | Least Angle Regression | 1.4967 | 3.558 | 1.8742 | 0.8373 | 0.0657 | 0.055 | 0.006 |
| male | FALSE | bmi | huber | Huber Regressor | 1.4975 | 3.5612 | 1.8751 | 0.8373 | 0.0656 | 0.0551 | 0.006 |
| male | FALSE | bmi | omp | Orthogonal Matching Pursuit | 1.5737 | 3.9199 | 1.9725 | 0.8231 | 0.0692 | 0.0579 | 0.006 |
| male | FALSE | bmi | gbr | Gradient Boosting Regressor | 1.6015 | 3.9178 | 1.9727 | 0.8231 | 0.0699 | 0.0592 | 0.011 |
| male | FALSE | bmi | ada | AdaBoost Regressor | 1.5974 | 3.9958 | 1.9848 | 0.8215 | 0.0703 | 0.0589 | 0.01 |
| male | FALSE | bmi | knn | K Neighbors Regressor | 1.6263 | 4.0219 | 1.9949 | 0.8192 | 0.0706 | 0.06 | 0.008 |
| male | FALSE | bmi | lightgbm | Light Gradient Boosting Machine | 1.656 | 4.2605 | 2.0554 | 0.807 | 0.0726 | 0.0611 | 0.028 |
| male | FALSE | bmi | rf | Random Forest Regressor | 1.7046 | 4.3249 | 2.0718 | 0.8038 | 0.0739 | 0.0633 | 0.031 |
| male | FALSE | bmi | et | Extra Trees Regressor | 1.7932 | 4.8691 | 2.2006 | 0.7793 | 0.0782 | 0.0664 | 0.026 |
| male | FALSE | bmi | xgboost | Extreme Gradient Boosting | 1.7856 | 5.0046 | 2.2299 | 0.7726 | 0.079 | 0.0662 | 0.021 |
| male | FALSE | bmi | par | Passive Aggressive Regressor | 1.8422 | 5.8358 | 2.2839 | 0.7185 | 0.0783 | 0.0679 | 0.007 |
| male | FALSE | bmi | dt | Decision Tree Regressor | 2.0921 | 6.7827 | 2.5977 | 0.6885 | 0.0927 | 0.0778 | 0.006 |
| male | FALSE | bmi | lasso | Lasso Regression | 3.8327 | 23.7941 | 4.8521 | -0.035 | 0.1684 | 0.1414 | 0.175 |
| male | FALSE | bmi | en | Elastic Net | 3.8327 | 23.7941 | 4.8521 | -0.035 | 0.1684 | 0.1414 | 0.006 |
| male | FALSE | bmi | llar | Lasso Least Angle Regression | 3.8327 | 23.7941 | 4.8521 | -0.035 | 0.1684 | 0.1414 | 0.006 |
| male | FALSE | bmi | dummy | Dummy Regressor | 3.8327 | 23.7941 | 4.8521 | -0.035 | 0.1684 | 0.1414 | 0.006 |
| male | TRUE | uralbcr | rf | Random Forest Regressor | 2.579 | 15.2952 | 3.826 | 0.027 | 0.4458 | 0.4299 | 0.047 |
| male | FALSE | uralbcr | lr | Linear Regression | 2.5468 | 15.3594 | 3.8423 | 0.0149 | 0.4403 | 0.4177 | 0.007 |
| male | FALSE | uralbcr | ridge | Ridge Regression | 2.5465 | 15.3596 | 3.8423 | 0.0149 | 0.4403 | 0.4176 | 0.007 |
| male | FALSE | uralbcr | lar | Least Angle Regression | 2.5468 | 15.3594 | 3.8423 | 0.0149 | 0.4403 | 0.4177 | 0.006 |
| male | FALSE | uralbcr | huber | Huber Regressor | 2.5507 | 15.3779 | 3.8447 | 0.0124 | 0.4415 | 0.4148 | 0.007 |
| male | FALSE | uralbcr | ada | AdaBoost Regressor | 2.5974 | 15.4346 | 3.854 | 0.0092 | 0.4463 | 0.4429 | 0.014 |
| male | FALSE | uralbcr | br | Bayesian Ridge | 2.5454 | 15.4929 | 3.859 | 0.0083 | 0.4402 | 0.4163 | 0.008 |
| male | FALSE | uralbcr | et | Extra Trees Regressor | 2.5962 | 15.6659 | 3.878 | -0.0025 | 0.4509 | 0.4302 | 0.029 |
| male | FALSE | uralbcr | xgboost | Extreme Gradient Boosting | 2.6897 | 15.6457 | 3.8829 | -0.0231 | 0.4727 | 0.4671 | 0.019 |
| male | FALSE | uralbcr | lightgbm | Light Gradient Boosting Machine | 2.7141 | 15.8446 | 3.9093 | -0.0249 | 0.4697 | 0.4688 | 0.037 |
| male | FALSE | uralbcr | gbr | Gradient Boosting Regressor | 2.6636 | 16.1117 | 3.9494 | -0.0517 | 0.4606 | 0.4481 | 0.019 |
| male | FALSE | uralbcr | omp | Orthogonal Matching Pursuit | 2.6326 | 16.4693 | 3.9823 | -0.0548 | 0.4591 | 0.4354 | 0.011 |
| male | FALSE | uralbcr | lasso | Lasso Regression | 2.651 | 17.1842 | 4.0648 | -0.0969 | 0.4671 | 0.4385 | 0.008 |
| male | FALSE | uralbcr | en | Elastic Net | 2.651 | 17.1842 | 4.0648 | -0.0969 | 0.4671 | 0.4385 | 0.007 |
| male | FALSE | uralbcr | llar | Lasso Least Angle Regression | 2.651 | 17.1842 | 4.0648 | -0.0969 | 0.4671 | 0.4385 | 0.006 |
| male | FALSE | uralbcr | dummy | Dummy Regressor | 2.651 | 17.1842 | 4.0648 | -0.0969 | 0.4671 | 0.4385 | 0.006 |
| male | FALSE | uralbcr | knn | K Neighbors Regressor | 2.7257 | 17.2957 | 4.0758 | -0.1094 | 0.4833 | 0.4537 | 0.009 |
| male | FALSE | uralbcr | dt | Decision Tree Regressor | 3.4736 | 24.252 | 4.8788 | -0.6718 | 0.5832 | 0.6514 | 0.006 |
| male | FALSE | uralbcr | par | Passive Aggressive Regressor | 3.9685 | 106.3974 | 7.5391 | -6.2231 | 0.5763 | 0.6874 | 0.008 |
| male | TRUE | uricacid | br | Bayesian Ridge | 0.0288 | 0.0013 | 0.0356 | 0.0996 | 0.0294 | 0.1436 | 0.006 |
| male | FALSE | uricacid | ridge | Ridge Regression | 0.0288 | 0.0013 | 0.0356 | 0.0991 | 0.0294 | 0.1436 | 0.005 |
| male | FALSE | uricacid | lr | Linear Regression | 0.0288 | 0.0013 | 0.0356 | 0.099 | 0.0294 | 0.1436 | 0.345 |
| male | FALSE | uricacid | lar | Least Angle Regression | 0.0288 | 0.0013 | 0.0356 | 0.099 | 0.0294 | 0.1436 | 0.006 |
| male | FALSE | uricacid | huber | Huber Regressor | 0.0289 | 0.0013 | 0.0356 | 0.0976 | 0.0294 | 0.1439 | 0.006 |
| male | FALSE | uricacid | omp | Orthogonal Matching Pursuit | 0.0289 | 0.0013 | 0.036 | 0.081 | 0.0297 | 0.144 | 0.006 |
| male | FALSE | uricacid | ada | AdaBoost Regressor | 0.0297 | 0.0013 | 0.0363 | 0.0612 | 0.03 | 0.148 | 0.008 |
| male | FALSE | uricacid | lasso | Lasso Regression | 0.0305 | 0.0015 | 0.0378 | -0.0065 | 0.0312 | 0.1519 | 0.154 |
| male | FALSE | uricacid | llar | Lasso Least Angle Regression | 0.0305 | 0.0015 | 0.0378 | -0.0065 | 0.0312 | 0.1519 | 0.006 |
| male | FALSE | uricacid | en | Elastic Net | 0.0305 | 0.0015 | 0.0378 | -0.0065 | 0.0312 | 0.1519 | 0.005 |
| male | FALSE | uricacid | dummy | Dummy Regressor | 0.0305 | 0.0015 | 0.0378 | -0.0065 | 0.0312 | 0.1519 | 0.005 |
| male | FALSE | uricacid | gbr | Gradient Boosting Regressor | 0.0312 | 0.0015 | 0.0383 | -0.0483 | 0.0316 | 0.1552 | 0.012 |
| male | FALSE | uricacid | lightgbm | Light Gradient Boosting Machine | 0.0316 | 0.0015 | 0.039 | -0.0854 | 0.0322 | 0.1571 | 0.028 |
| male | FALSE | uricacid | knn | K Neighbors Regressor | 0.0319 | 0.0016 | 0.0394 | -0.1054 | 0.0325 | 0.1585 | 0.008 |
| male | FALSE | uricacid | rf | Random Forest Regressor | 0.0328 | 0.0016 | 0.0398 | -0.1444 | 0.0328 | 0.1625 | 0.031 |
| male | FALSE | uricacid | et | Extra Trees Regressor | 0.0332 | 0.0017 | 0.0405 | -0.187 | 0.0334 | 0.1644 | 0.023 |
| male | FALSE | uricacid | xgboost | Extreme Gradient Boosting | 0.0352 | 0.0019 | 0.0437 | -0.3684 | 0.036 | 0.1745 | 0.021 |
| male | FALSE | uricacid | par | Passive Aggressive Regressor | 0.0376 | 0.0021 | 0.0458 | -0.6378 | 0.0379 | 0.1813 | 0.006 |
| male | FALSE | uricacid | dt | Decision Tree Regressor | 0.0427 | 0.0027 | 0.0517 | -0.9664 | 0.0426 | 0.21 | 0.006 |
| male | TRUE | bloodglucose | huber | Huber Regressor | 0.4165 | 0.2825 | 0.5296 | 0.1092 | 0.0798 | 0.0749 | 0.006 |
| male | FALSE | bloodglucose | lr | Linear Regression | 0.4161 | 0.283 | 0.5298 | 0.109 | 0.0798 | 0.0747 | 0.31 |
| male | FALSE | bloodglucose | lar | Least Angle Regression | 0.4161 | 0.283 | 0.5298 | 0.109 | 0.0798 | 0.0747 | 0.006 |
| male | FALSE | bloodglucose | ridge | Ridge Regression | 0.4161 | 0.283 | 0.5298 | 0.1089 | 0.0798 | 0.0747 | 0.006 |
| male | FALSE | bloodglucose | br | Bayesian Ridge | 0.4159 | 0.2835 | 0.5302 | 0.108 | 0.0799 | 0.0746 | 0.006 |
| male | FALSE | bloodglucose | omp | Orthogonal Matching Pursuit | 0.427 | 0.3049 | 0.5501 | 0.0396 | 0.0827 | 0.0764 | 0.012 |
| male | FALSE | bloodglucose | ada | AdaBoost Regressor | 0.4426 | 0.3209 | 0.5638 | -0.0076 | 0.0849 | 0.0792 | 0.012 |
| male | FALSE | bloodglucose | lasso | Lasso Regression | 0.4547 | 0.3434 | 0.5828 | -0.0728 | 0.0875 | 0.0812 | 0.148 |
| male | FALSE | bloodglucose | llar | Lasso Least Angle Regression | 0.4547 | 0.3434 | 0.5828 | -0.0728 | 0.0875 | 0.0812 | 0.006 |
| male | FALSE | bloodglucose | en | Elastic Net | 0.4547 | 0.3434 | 0.5828 | -0.0728 | 0.0875 | 0.0812 | 0.006 |
| male | FALSE | bloodglucose | dummy | Dummy Regressor | 0.4547 | 0.3434 | 0.5828 | -0.0728 | 0.0875 | 0.0812 | 0.005 |
| male | FALSE | bloodglucose | rf | Random Forest Regressor | 0.4594 | 0.3405 | 0.5816 | -0.08 | 0.0877 | 0.0824 | 0.036 |
| male | FALSE | bloodglucose | lightgbm | Light Gradient Boosting Machine | 0.4641 | 0.3474 | 0.5865 | -0.0987 | 0.0887 | 0.0835 | 0.03 |
| male | FALSE | bloodglucose | et | Extra Trees Regressor | 0.4699 | 0.3557 | 0.5933 | -0.1217 | 0.0894 | 0.0843 | 0.025 |
| male | FALSE | bloodglucose | gbr | Gradient Boosting Regressor | 0.4714 | 0.3588 | 0.5977 | -0.1425 | 0.0901 | 0.0846 | 0.013 |
| male | FALSE | bloodglucose | knn | K Neighbors Regressor | 0.4709 | 0.3599 | 0.5978 | -0.1435 | 0.0904 | 0.0847 | 0.008 |
| male | FALSE | bloodglucose | xgboost | Extreme Gradient Boosting | 0.4795 | 0.3789 | 0.6136 | -0.208 | 0.0927 | 0.0864 | 0.022 |
| male | FALSE | bloodglucose | dt | Decision Tree Regressor | 0.6178 | 0.6146 | 0.7799 | -0.9839 | 0.1173 | 0.1109 | 0.006 |
| male | FALSE | bloodglucose | par | Passive Aggressive Regressor | 1.9962 | 80.6497 | 4.8021 | -279.6765 | 0.3273 | 0.3583 | 0.006 |
| male | TRUE | hdl | lr | Linear Regression | 0.1963 | 0.065 | 0.2535 | 0.1844 | 0.1072 | 0.1529 | 0.348 |
| male | FALSE | hdl | lar | Least Angle Regression | 0.1963 | 0.065 | 0.2535 | 0.1844 | 0.1072 | 0.1529 | 0.006 |
| male | FALSE | hdl | ridge | Ridge Regression | 0.1963 | 0.065 | 0.2535 | 0.1843 | 0.1072 | 0.1528 | 0.006 |
| male | FALSE | hdl | br | Bayesian Ridge | 0.1961 | 0.0651 | 0.2538 | 0.1827 | 0.1073 | 0.1526 | 0.006 |
| male | FALSE | hdl | huber | Huber Regressor | 0.1967 | 0.0653 | 0.2542 | 0.1798 | 0.1075 | 0.1531 | 0.006 |
| male | FALSE | hdl | rf | Random Forest Regressor | 0.2041 | 0.0695 | 0.2617 | 0.13 | 0.1112 | 0.1602 | 0.04 |
| male | FALSE | hdl | ada | AdaBoost Regressor | 0.2068 | 0.0697 | 0.2631 | 0.1175 | 0.1116 | 0.1608 | 0.011 |
| male | FALSE | hdl | omp | Orthogonal Matching Pursuit | 0.2031 | 0.0713 | 0.2656 | 0.1042 | 0.1121 | 0.1578 | 0.005 |
| male | FALSE | hdl | gbr | Gradient Boosting Regressor | 0.2075 | 0.0713 | 0.2654 | 0.1027 | 0.1125 | 0.1626 | 0.013 |
| male | FALSE | hdl | et | Extra Trees Regressor | 0.2143 | 0.0747 | 0.2714 | 0.0652 | 0.1154 | 0.1681 | 0.03 |
| male | FALSE | hdl | lightgbm | Light Gradient Boosting Machine | 0.2184 | 0.0779 | 0.2771 | 0.0204 | 0.1183 | 0.1721 | 0.036 |
| male | FALSE | hdl | knn | K Neighbors Regressor | 0.219 | 0.0798 | 0.2811 | -0.008 | 0.1192 | 0.1702 | 0.008 |
| male | FALSE | hdl | lasso | Lasso Regression | 0.2195 | 0.0823 | 0.2855 | -0.0321 | 0.1206 | 0.1706 | 0.152 |
| male | FALSE | hdl | en | Elastic Net | 0.2195 | 0.0823 | 0.2855 | -0.0321 | 0.1206 | 0.1706 | 0.006 |
| male | FALSE | hdl | llar | Lasso Least Angle Regression | 0.2195 | 0.0823 | 0.2855 | -0.0321 | 0.1206 | 0.1706 | 0.006 |
| male | FALSE | hdl | dummy | Dummy Regressor | 0.2195 | 0.0823 | 0.2855 | -0.0321 | 0.1206 | 0.1706 | 0.006 |
| male | FALSE | hdl | xgboost | Extreme Gradient Boosting | 0.2284 | 0.0877 | 0.2936 | -0.1058 | 0.1248 | 0.1805 | 0.033 |
| male | FALSE | hdl | par | Passive Aggressive Regressor | 0.2363 | 0.0938 | 0.3032 | -0.1661 | 0.1291 | 0.1831 | 0.006 |
| male | FALSE | hdl | dt | Decision Tree Regressor | 0.2963 | 0.1402 | 0.3736 | -0.8078 | 0.1584 | 0.2359 | 0.006 |
| male | TRUE | triglycerides | et | Extra Trees Regressor | 0.372 | 0.2306 | 0.4779 | 0.1482 | 0.2014 | 0.3285 | 0.031 |
| male | FALSE | triglycerides | lr | Linear Regression | 0.3672 | 0.2327 | 0.4803 | 0.1429 | 0.2023 | 0.3217 | 0.323 |
| male | FALSE | triglycerides | ridge | Ridge Regression | 0.3673 | 0.2327 | 0.4803 | 0.1429 | 0.2023 | 0.3218 | 0.006 |
| male | FALSE | triglycerides | lar | Least Angle Regression | 0.3672 | 0.2327 | 0.4803 | 0.1429 | 0.2023 | 0.3217 | 0.006 |
| male | FALSE | triglycerides | br | Bayesian Ridge | 0.3689 | 0.2343 | 0.4821 | 0.1383 | 0.2028 | 0.3238 | 0.006 |
| male | FALSE | triglycerides | rf | Random Forest Regressor | 0.3752 | 0.2351 | 0.4829 | 0.1316 | 0.204 | 0.3326 | 0.063 |
| male | FALSE | triglycerides | huber | Huber Regressor | 0.367 | 0.2358 | 0.4833 | 0.1311 | 0.2033 | 0.3161 | 0.006 |
| male | FALSE | triglycerides | ada | AdaBoost Regressor | 0.3876 | 0.2503 | 0.4987 | 0.0761 | 0.2119 | 0.3523 | 0.012 |
| male | FALSE | triglycerides | omp | Orthogonal Matching Pursuit | 0.3846 | 0.2534 | 0.5012 | 0.0758 | 0.2113 | 0.3422 | 0.006 |
| male | FALSE | triglycerides | gbr | Gradient Boosting Regressor | 0.3831 | 0.2527 | 0.5015 | 0.0589 | 0.212 | 0.3399 | 0.015 |
| male | FALSE | triglycerides | lightgbm | Light Gradient Boosting Machine | 0.3916 | 0.2558 | 0.5037 | 0.0552 | 0.2137 | 0.3463 | 0.033 |
| male | FALSE | triglycerides | knn | K Neighbors Regressor | 0.4034 | 0.2643 | 0.5125 | 0.0242 | 0.2183 | 0.3618 | 0.008 |
| male | FALSE | triglycerides | xgboost | Extreme Gradient Boosting | 0.4018 | 0.2676 | 0.5159 | 0.0116 | 0.2189 | 0.3571 | 0.029 |
| male | FALSE | triglycerides | lasso | Lasso Regression | 0.4192 | 0.2919 | 0.5372 | -0.0538 | 0.2275 | 0.3777 | 0.151 |
| male | FALSE | triglycerides | en | Elastic Net | 0.4192 | 0.2919 | 0.5372 | -0.0538 | 0.2275 | 0.3777 | 0.006 |
| male | FALSE | triglycerides | llar | Lasso Least Angle Regression | 0.4192 | 0.2919 | 0.5372 | -0.0538 | 0.2275 | 0.3777 | 0.006 |
| male | FALSE | triglycerides | dummy | Dummy Regressor | 0.4192 | 0.2919 | 0.5372 | -0.0538 | 0.2275 | 0.3777 | 0.006 |
| male | FALSE | triglycerides | dt | Decision Tree Regressor | 0.5521 | 0.4898 | 0.699 | -0.8395 | 0.297 | 0.5122 | 0.006 |
| male | FALSE | triglycerides | par | Passive Aggressive Regressor | 0.5454 | 2.0797 | 0.9719 | -6.3693 | 0.2833 | 0.4795 | 0.006 |
| male | TRUE | albuminuria | br | Bayesian Ridge | 0.2018 | 0.0645 | 0.2536 | 0.194 | 0.0477 | 0.0468 | 0.007 |
| male | FALSE | albuminuria | ridge | Ridge Regression | 0.2021 | 0.0647 | 0.2541 | 0.1902 | 0.0477 | 0.0468 | 0.006 |
| male | FALSE | albuminuria | lr | Linear Regression | 0.2022 | 0.0647 | 0.2541 | 0.1899 | 0.0477 | 0.0468 | 0.351 |
| male | FALSE | albuminuria | lar | Least Angle Regression | 0.2022 | 0.0647 | 0.2541 | 0.1899 | 0.0477 | 0.0468 | 0.008 |
| male | FALSE | albuminuria | huber | Huber Regressor | 0.2025 | 0.0651 | 0.2548 | 0.1862 | 0.0479 | 0.047 | 0.007 |
| male | FALSE | albuminuria | ada | AdaBoost Regressor | 0.2062 | 0.068 | 0.2603 | 0.1442 | 0.0489 | 0.0478 | 0.014 |
| male | FALSE | albuminuria | omp | Orthogonal Matching Pursuit | 0.2071 | 0.0692 | 0.2627 | 0.138 | 0.0493 | 0.048 | 0.006 |
| male | FALSE | albuminuria | rf | Random Forest Regressor | 0.2134 | 0.071 | 0.2661 | 0.1107 | 0.0499 | 0.0494 | 0.045 |
| male | FALSE | albuminuria | et | Extra Trees Regressor | 0.2162 | 0.0728 | 0.2696 | 0.0909 | 0.0506 | 0.0501 | 0.033 |
| male | FALSE | albuminuria | gbr | Gradient Boosting Regressor | 0.2156 | 0.072 | 0.2675 | 0.0896 | 0.0503 | 0.0498 | 0.017 |
| male | FALSE | albuminuria | knn | K Neighbors Regressor | 0.2196 | 0.0751 | 0.2733 | 0.0646 | 0.0512 | 0.0508 | 0.017 |
| male | FALSE | albuminuria | en | Elastic Net | 0.2185 | 0.0766 | 0.2758 | 0.0599 | 0.0518 | 0.0507 | 0.007 |
| male | FALSE | albuminuria | lightgbm | Light Gradient Boosting Machine | 0.2224 | 0.0773 | 0.277 | 0.0347 | 0.0519 | 0.0514 | 0.033 |
| male | FALSE | albuminuria | lasso | Lasso Regression | 0.2277 | 0.0828 | 0.287 | -0.0165 | 0.0538 | 0.0527 | 0.152 |
| male | FALSE | albuminuria | llar | Lasso Least Angle Regression | 0.2277 | 0.0828 | 0.287 | -0.0165 | 0.0538 | 0.0527 | 0.006 |
| male | FALSE | albuminuria | dummy | Dummy Regressor | 0.2296 | 0.084 | 0.2891 | -0.0307 | 0.0542 | 0.0532 | 0.006 |
| male | FALSE | albuminuria | xgboost | Extreme Gradient Boosting | 0.2334 | 0.084 | 0.289 | -0.0477 | 0.0542 | 0.0539 | 0.029 |
| male | FALSE | albuminuria | dt | Decision Tree Regressor | 0.2817 | 0.1219 | 0.3483 | -0.5378 | 0.0654 | 0.0649 | 0.008 |
| male | FALSE | albuminuria | par | Passive Aggressive Regressor | 0.2926 | 0.1362 | 0.3637 | -0.7622 | 0.0684 | 0.067 | 0.007 |
| male | TRUE | sys_bp | huber | Huber Regressor | 9.6615 | 154.7346 | 12.3616 | 0.1117 | 0.0987 | 0.0787 | 0.014 |
| male | FALSE | sys_bp | br | Bayesian Ridge | 9.6537 | 155.1253 | 12.3763 | 0.1097 | 0.0988 | 0.0785 | 0.006 |
| male | FALSE | sys_bp | ridge | Ridge Regression | 9.6566 | 155.1348 | 12.3769 | 0.1096 | 0.0988 | 0.0786 | 0.006 |
| male | FALSE | sys_bp | lr | Linear Regression | 9.657 | 155.1401 | 12.3772 | 0.1095 | 0.0988 | 0.0786 | 0.324 |
| male | FALSE | sys_bp | lar | Least Angle Regression | 9.657 | 155.1401 | 12.3772 | 0.1095 | 0.0988 | 0.0786 | 0.006 |
| male | FALSE | sys_bp | ada | AdaBoost Regressor | 9.7514 | 159.7314 | 12.5701 | 0.08 | 0.1003 | 0.0793 | 0.013 |
| male | FALSE | sys_bp | rf | Random Forest Regressor | 10.0948 | 162.9794 | 12.679 | 0.0612 | 0.1011 | 0.0819 | 0.039 |
| male | FALSE | sys_bp | et | Extra Trees Regressor | 10.136 | 165.4455 | 12.7942 | 0.0459 | 0.102 | 0.0823 | 0.026 |
| male | FALSE | sys_bp | gbr | Gradient Boosting Regressor | 10.3005 | 168.1189 | 12.9093 | 0.022 | 0.1029 | 0.0836 | 0.013 |
| male | FALSE | sys_bp | knn | K Neighbors Regressor | 10.1973 | 171.1695 | 12.9947 | 0.0155 | 0.1039 | 0.0829 | 0.008 |
| male | FALSE | sys_bp | omp | Orthogonal Matching Pursuit | 10.1905 | 175.2232 | 13.1477 | -0.0027 | 0.1049 | 0.0828 | 0.006 |
| male | FALSE | sys_bp | lasso | Lasso Regression | 10.3562 | 180.9465 | 13.3608 | -0.0349 | 0.1065 | 0.084 | 0.149 |
| male | FALSE | sys_bp | en | Elastic Net | 10.3562 | 180.9465 | 13.3608 | -0.0349 | 0.1065 | 0.084 | 0.007 |
| male | FALSE | sys_bp | llar | Lasso Least Angle Regression | 10.3562 | 180.9465 | 13.3608 | -0.0349 | 0.1065 | 0.084 | 0.006 |
| male | FALSE | sys_bp | dummy | Dummy Regressor | 10.3562 | 180.9465 | 13.3608 | -0.0349 | 0.1065 | 0.084 | 0.006 |
| male | FALSE | sys_bp | lightgbm | Light Gradient Boosting Machine | 10.5986 | 179.3735 | 13.3152 | -0.0394 | 0.1062 | 0.0861 | 0.038 |
| male | FALSE | sys_bp | xgboost | Extreme Gradient Boosting | 10.4332 | 179.2919 | 13.3205 | -0.0403 | 0.1061 | 0.0845 | 0.019 |
| male | FALSE | sys_bp | dt | Decision Tree Regressor | 14.5165 | 331.3034 | 18.0663 | -0.9574 | 0.1452 | 0.1182 | 0.006 |

**Comments:**

During the training of both male and female models, we systematically evaluated the performance of various machine learning models with Recursive Feature Elimination with Cross-Balidation (RFECV). Our analysis revealed that Lasso Regression and Elastic Net consistently ranked among the lowest performing models in terms of their ability to generalise to test sets for both male and female datasets (Table 4).

Furthermore, we observed that Decision Tree Regressor, Elastic Net, and Lasso Regression often underperformed compared to boosted models such as Gradient Boosting Regression, and XGBoost. These findings suggest that the application RFECV did not universally enhance model performance and, in some cases, may have negatively impacted it.

**Potential Explanations:**

1. Certain models, such as Lasso Regression and Elastic Net, inherently perform feature selection through their regularization mechanisms. Applying an external feature selection method like RFECV may therefore provide limited or even detrimental benefits, as it could remove features that these models would have naturally downweighted or eliminated.
2. The impact of removing features may vary depending on feature correlations. RFECV eliminates features iteratively based on their individual importance 𝜷, which may inadvertently remove relevant information that contributes directly or indirectly to the variance in the training data. This could explain why Decision Tree-based models and regularized regression models struggled when subjected to RFECV frameworks in this study.

**Final thoughts:**

Our results demonstrate that while RFECV can be beneficial for certain model types, its effectiveness is model-dependent. Notably, models with built-in feature selection mechanisms, such as Lasso Regression and Elastic Net did not benefit from RFECV and instead performed worse than more complex models like XGBoost and Gradient Boosting Regression. These findings emphasize the need for model-specific feature selection strategies rather than a one-size-fits-all approach which is what our overall research aims to capture within a biological context.

**Supplementary Figure 1:** Showing the box plot results for the female model predicting the various biomarkers for the female and male cohort for each of the 9 biomarkers.


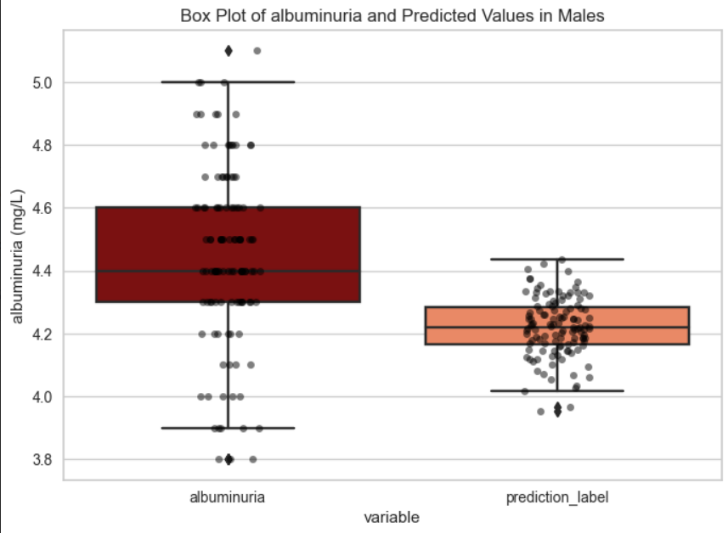

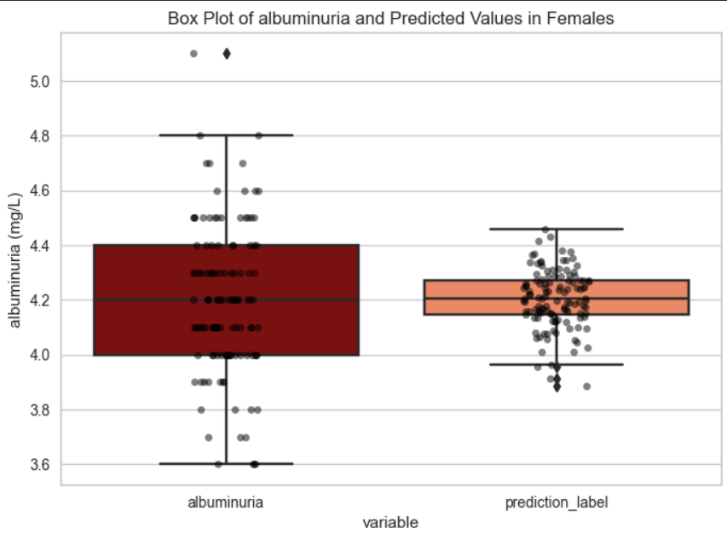

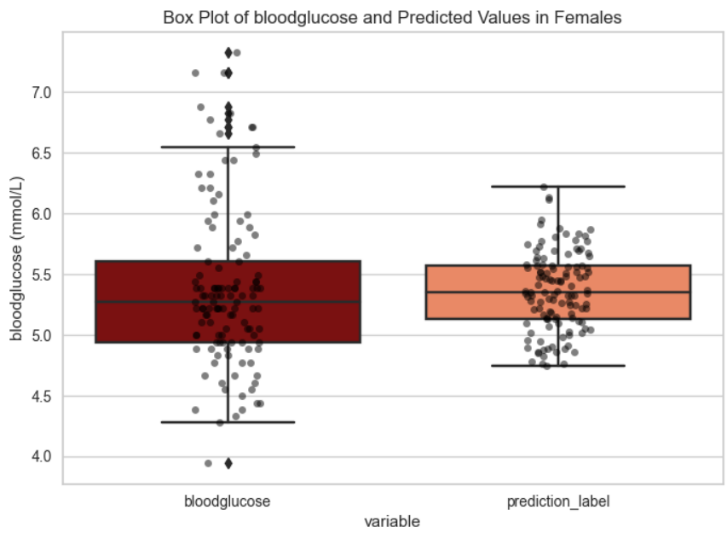

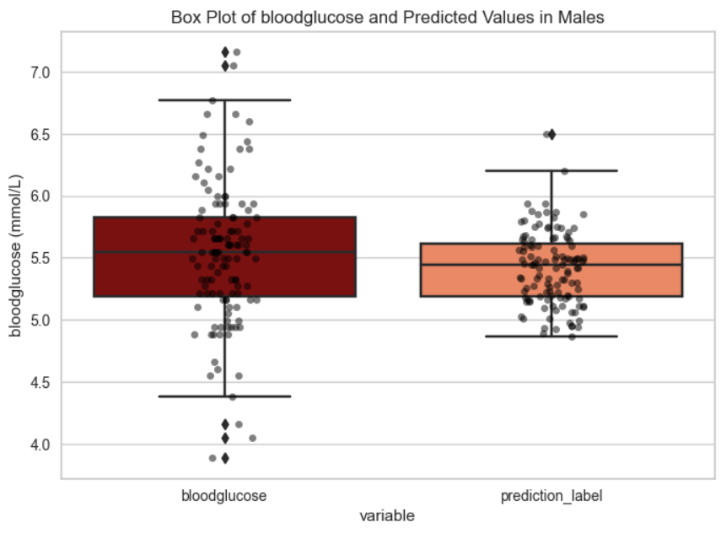

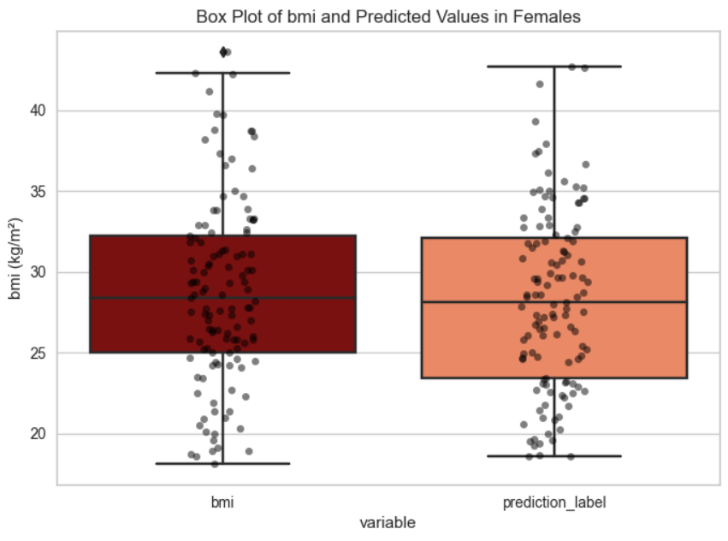

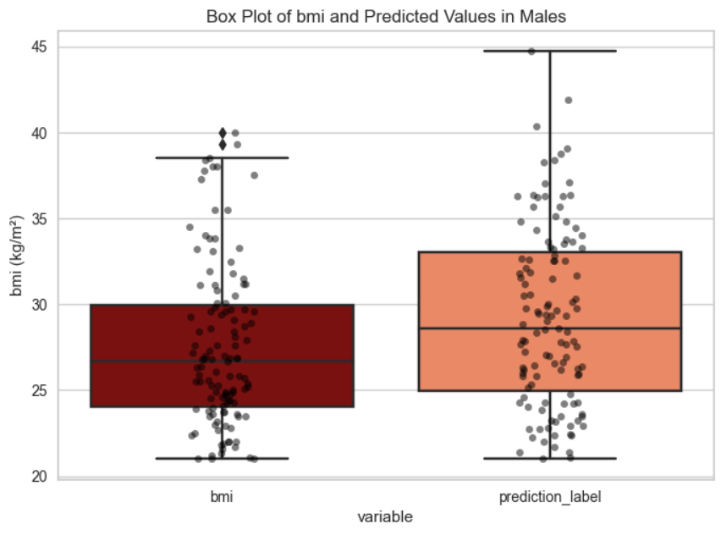

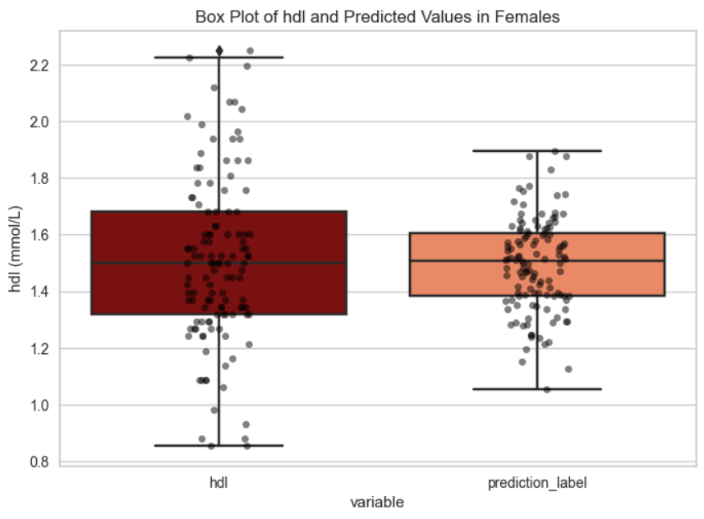

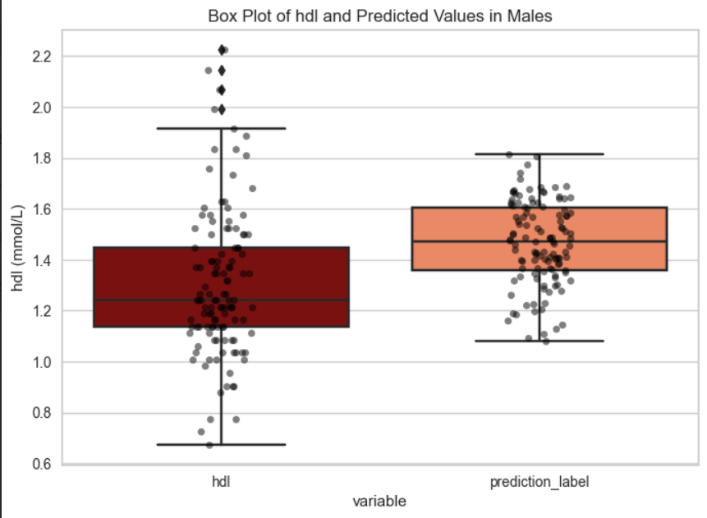

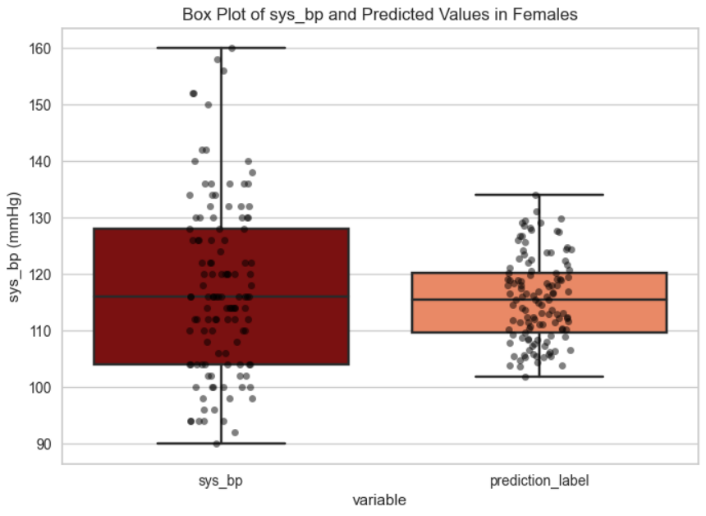

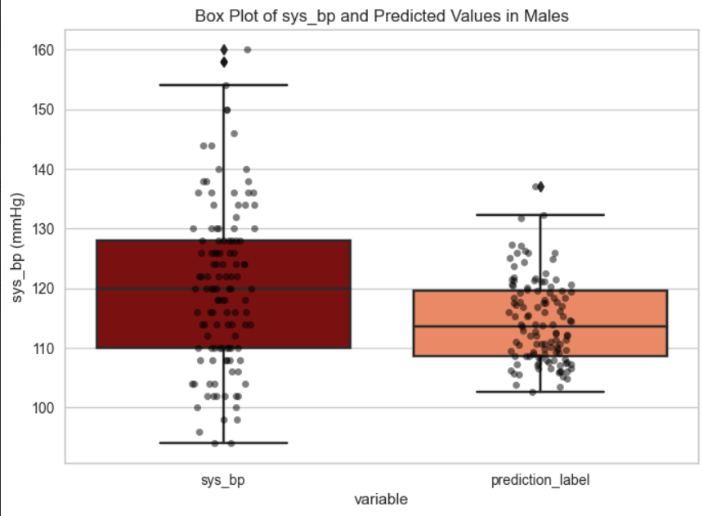

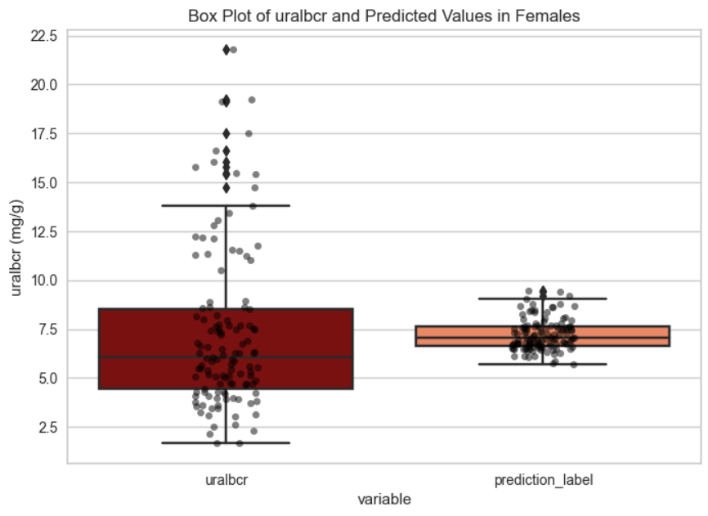

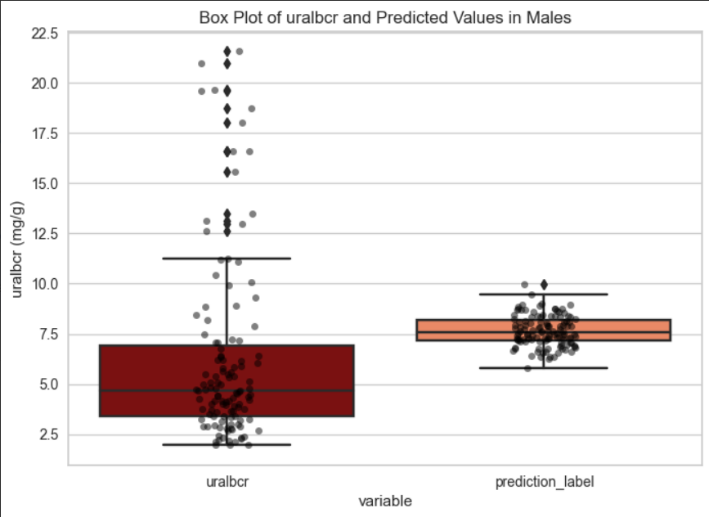

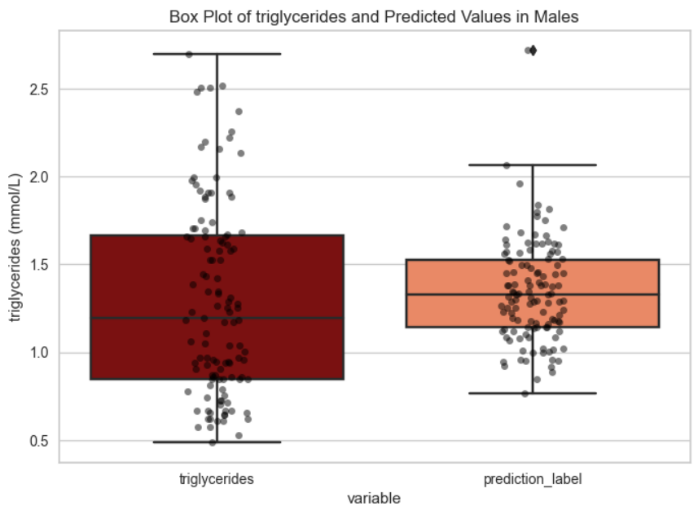

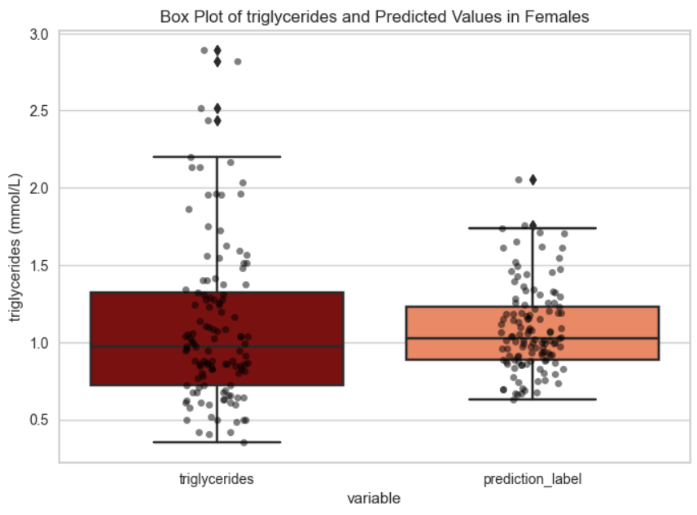

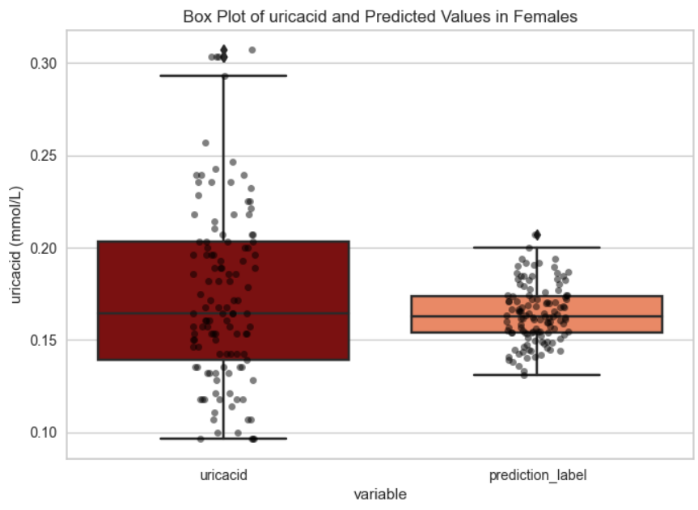

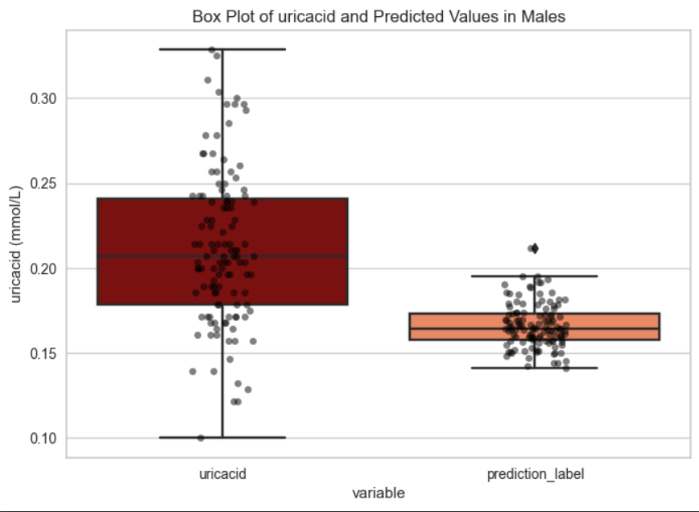

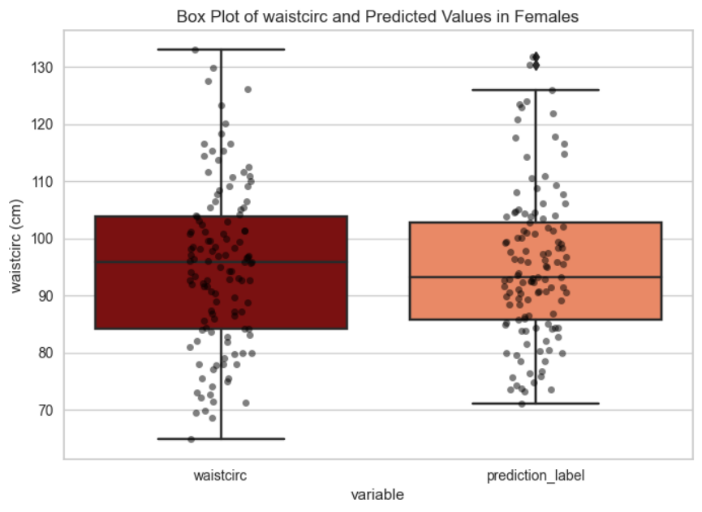

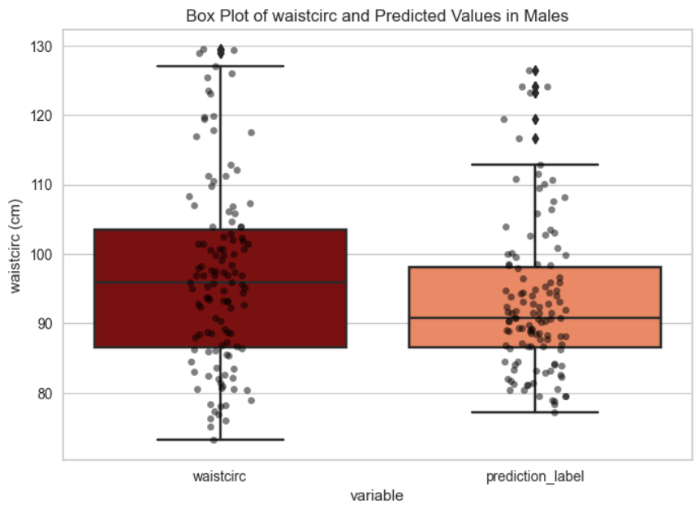


**Supplementary Table 2:** A summary table presenting the performance of the optimal female model on both male and female data partitions, including corresponding p-values from t-tests.


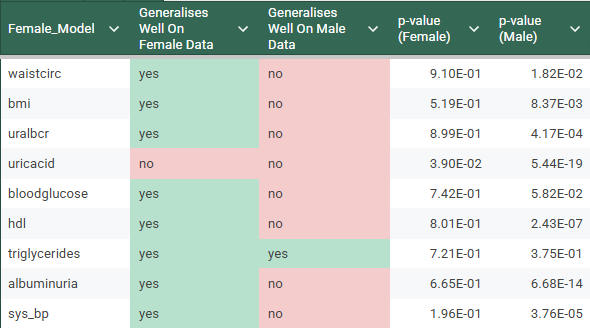


**Description:** H₀: The female-trained model predicted values without significant error. Hₐ: The female-trained model predicts values with significant error. H₀ is rejected if p < 0.05.


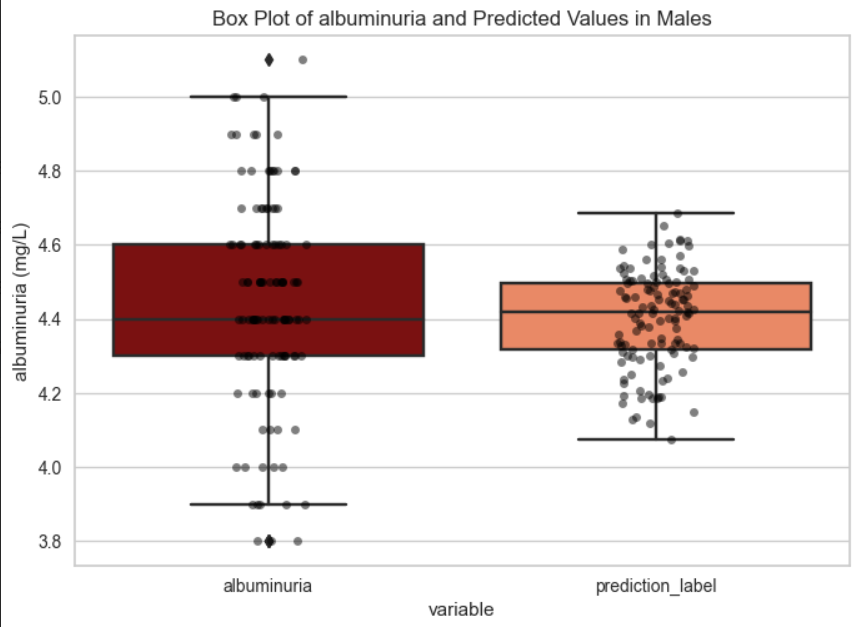

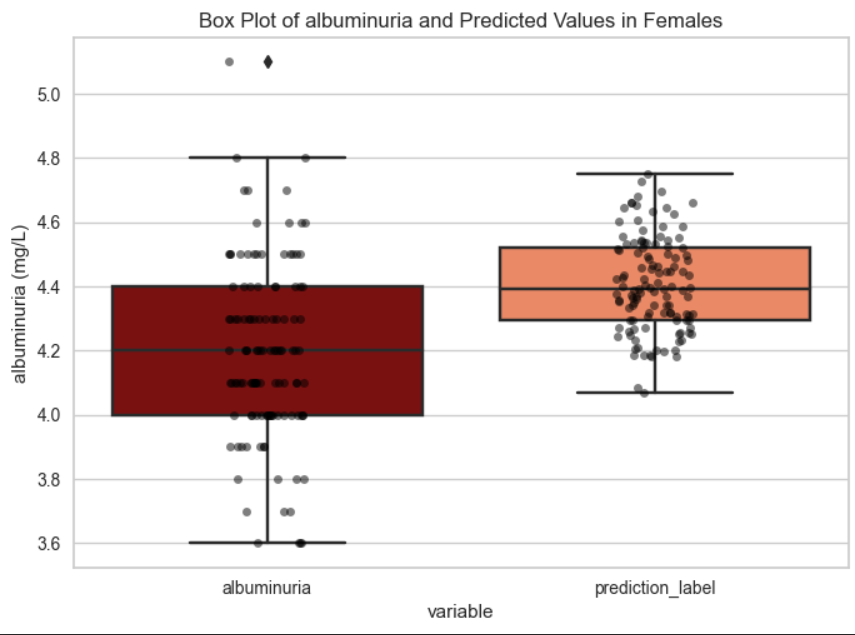
**Supplementary Figure 2:** Showing the box plot results for the male model predicting the various biomarkers for the male cohort and for the female cohort for each of the 9 biomarkers.


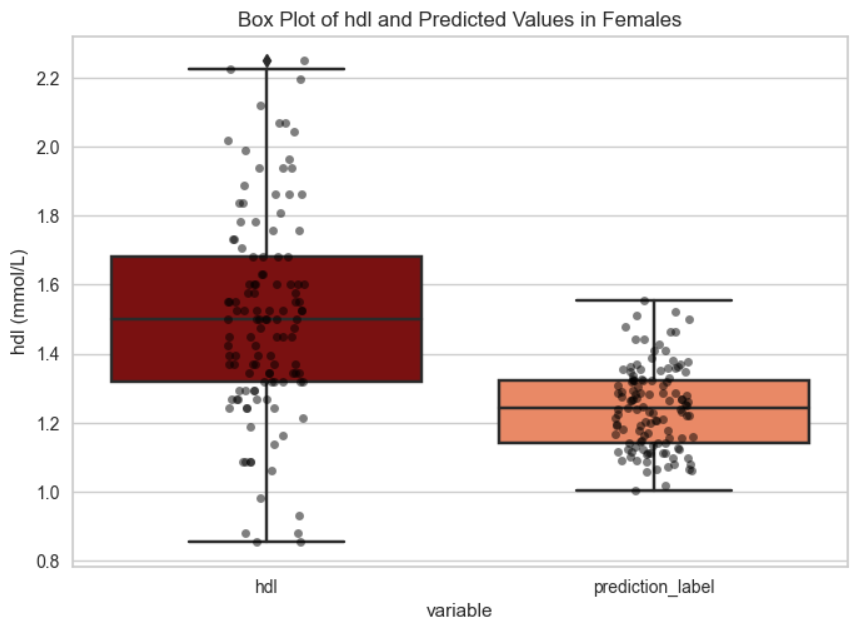

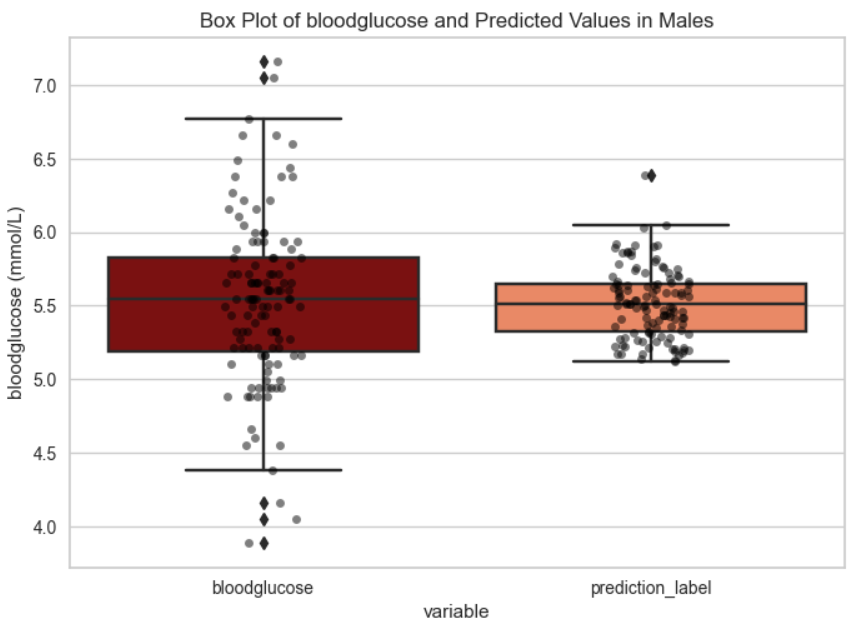

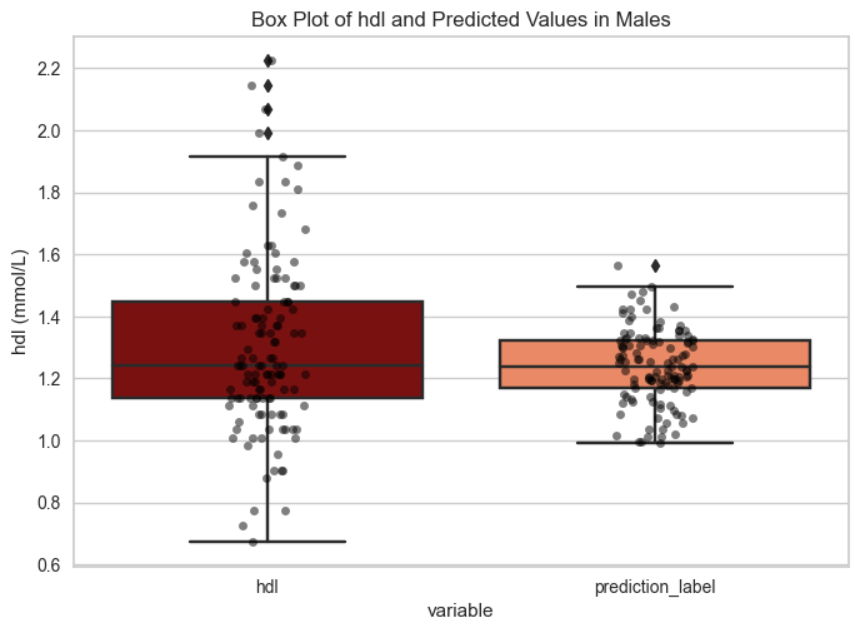

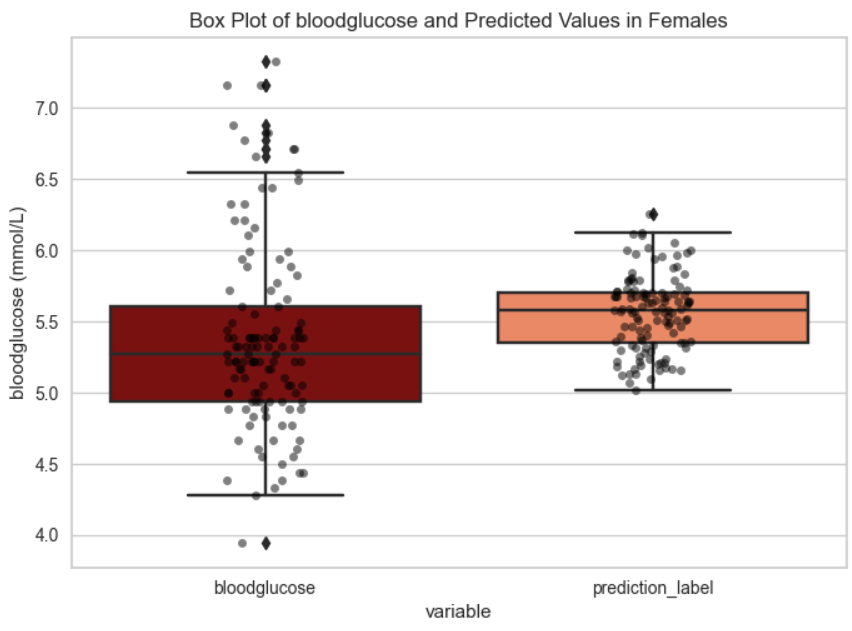

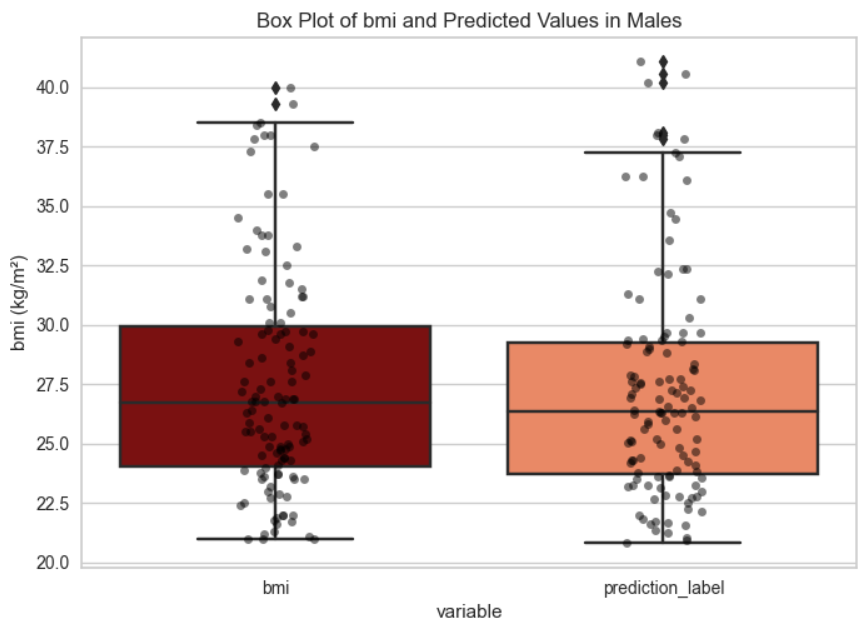

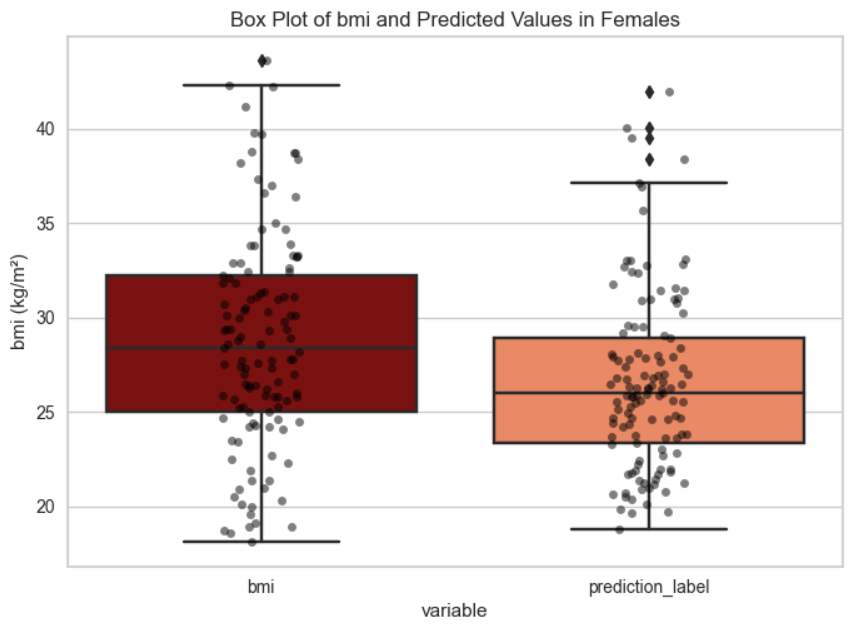


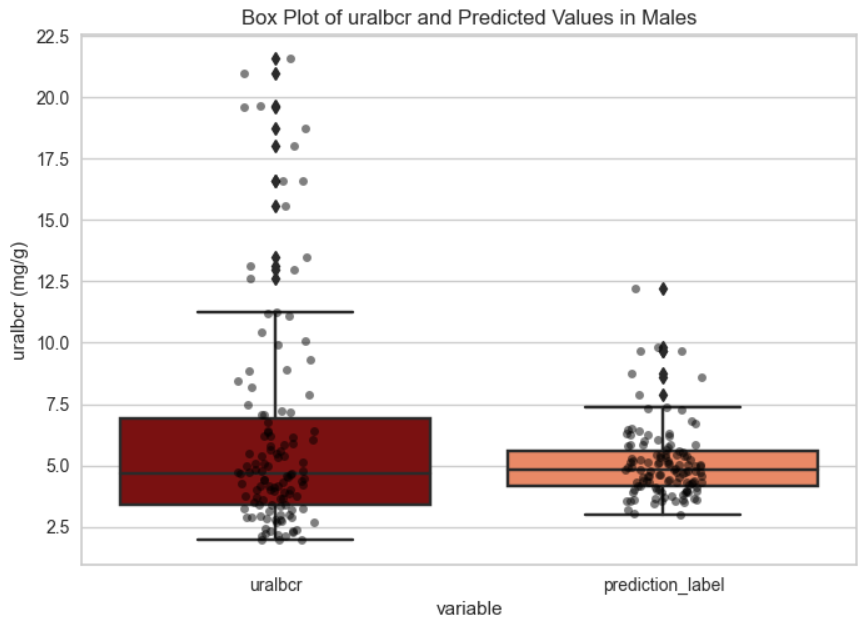

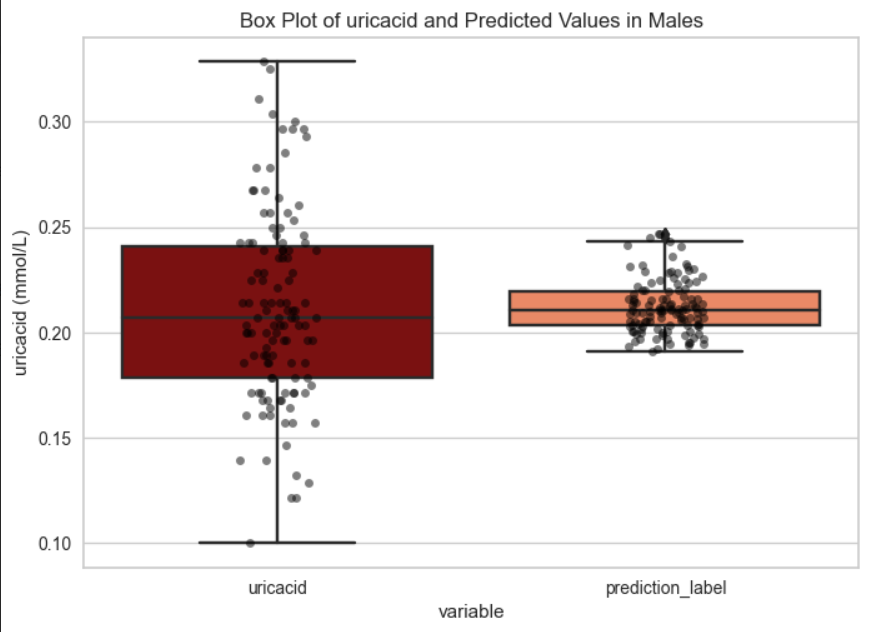

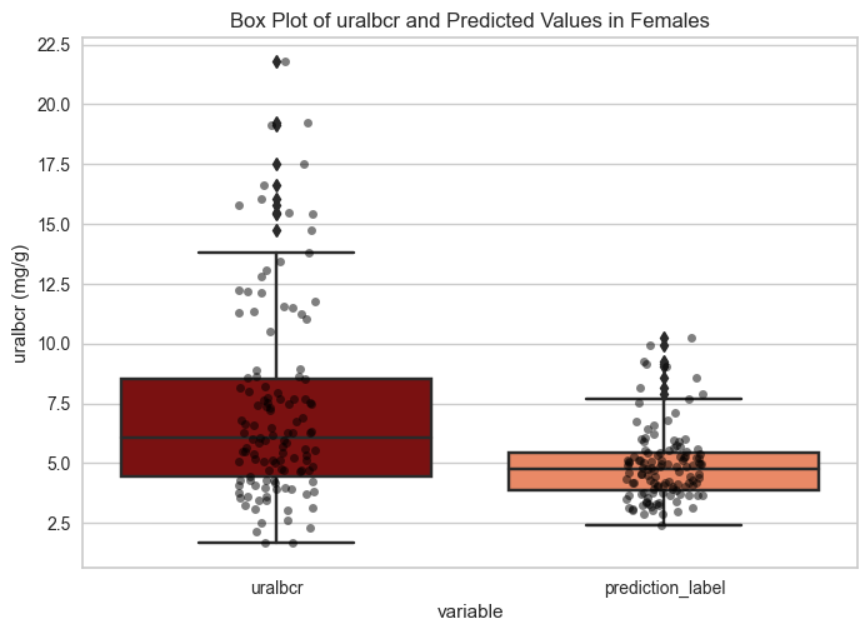

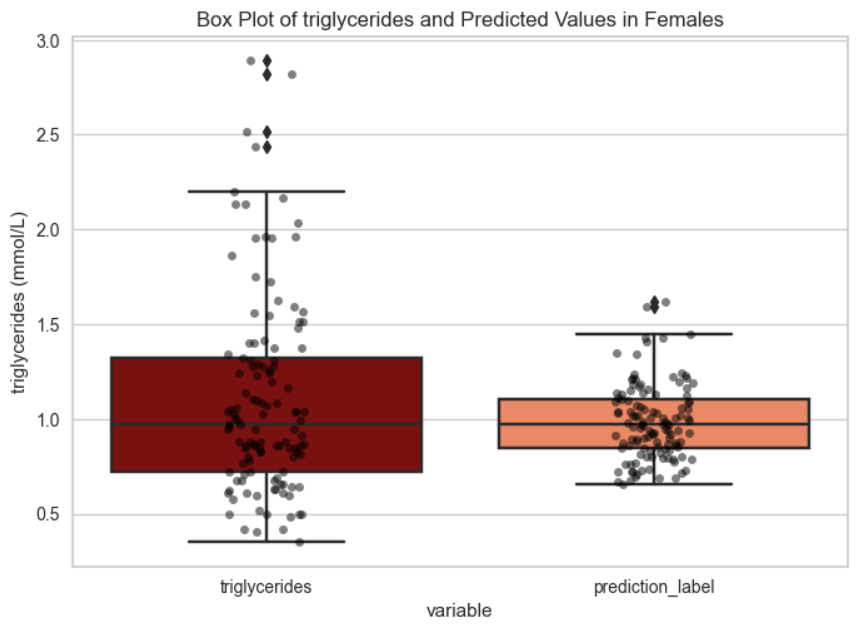

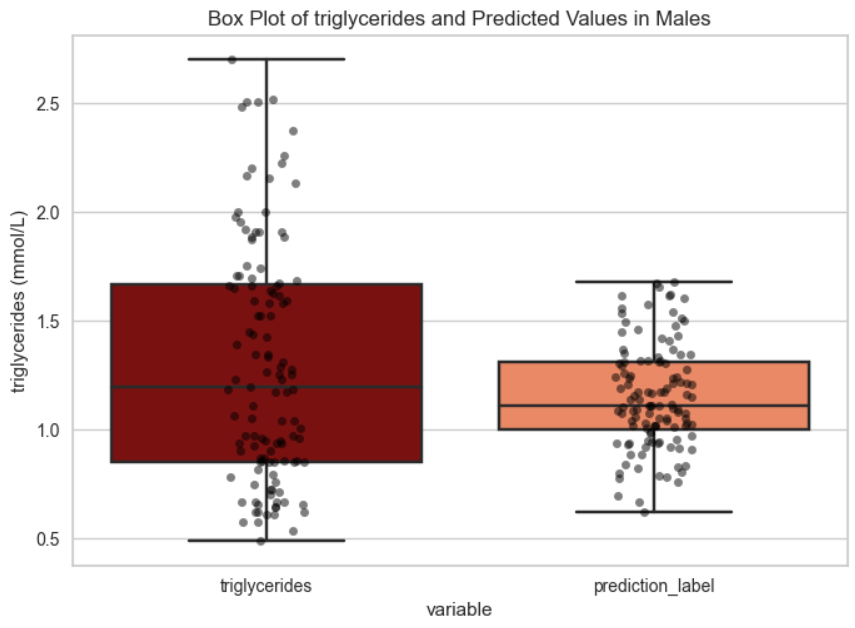

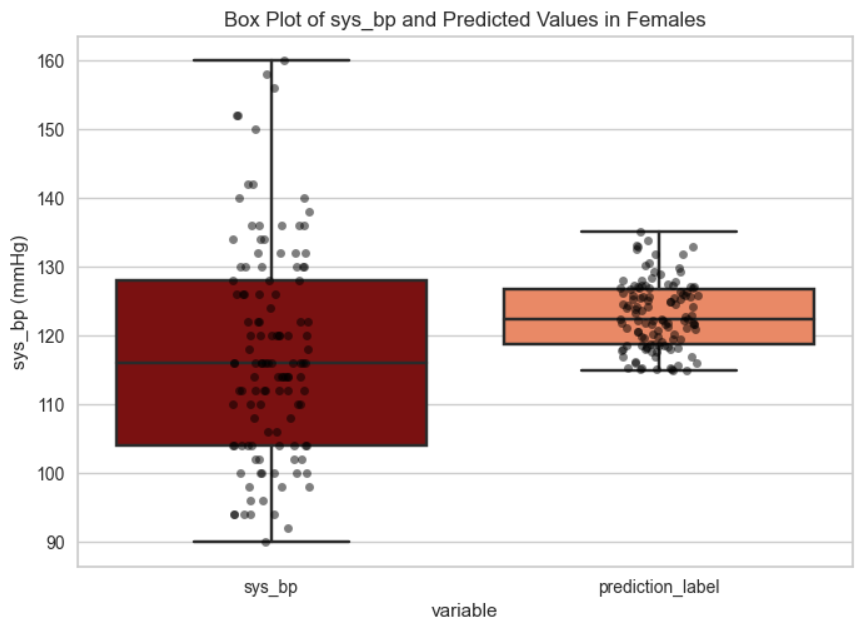

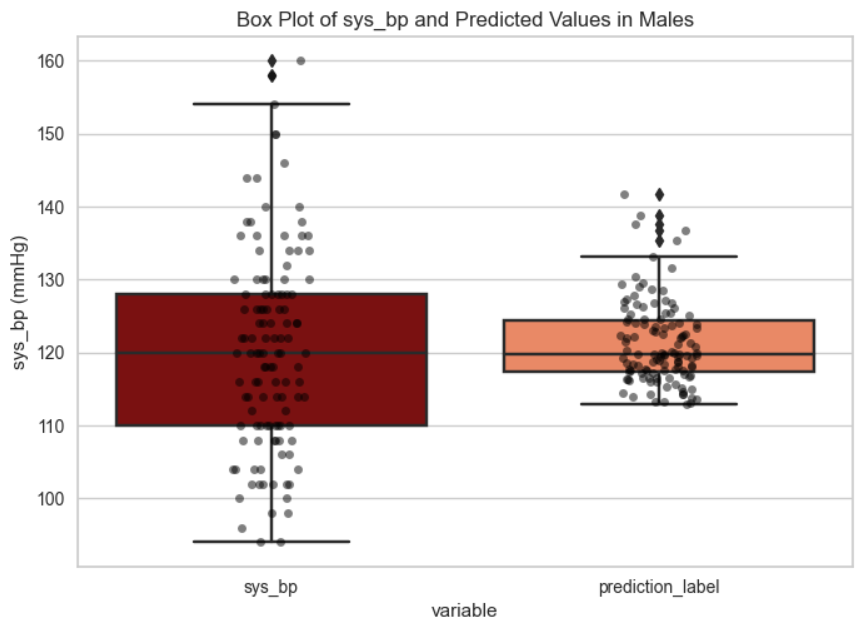

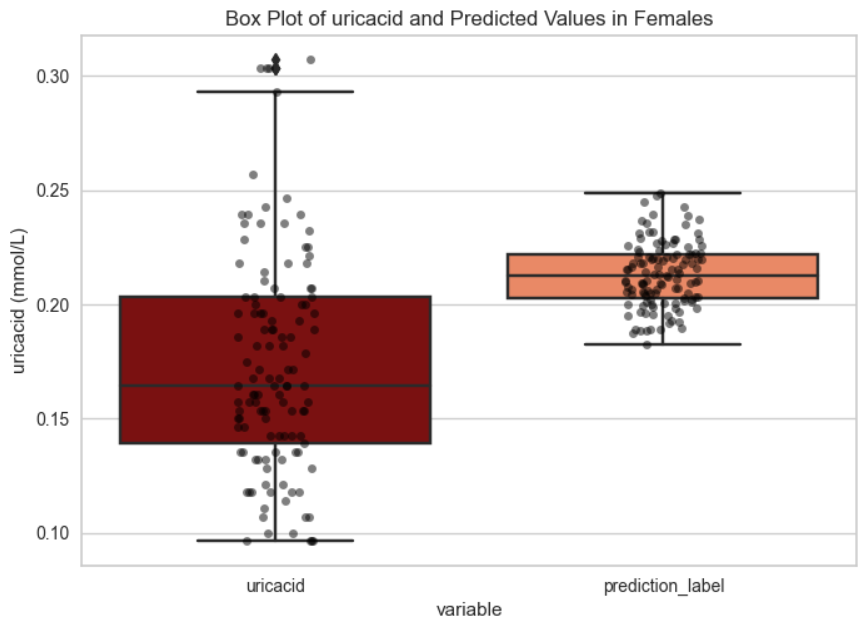


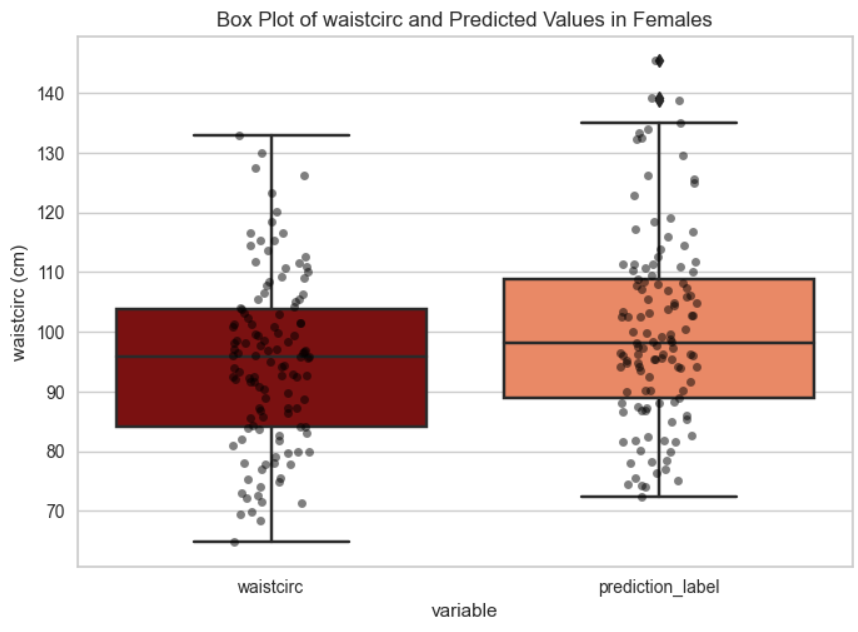

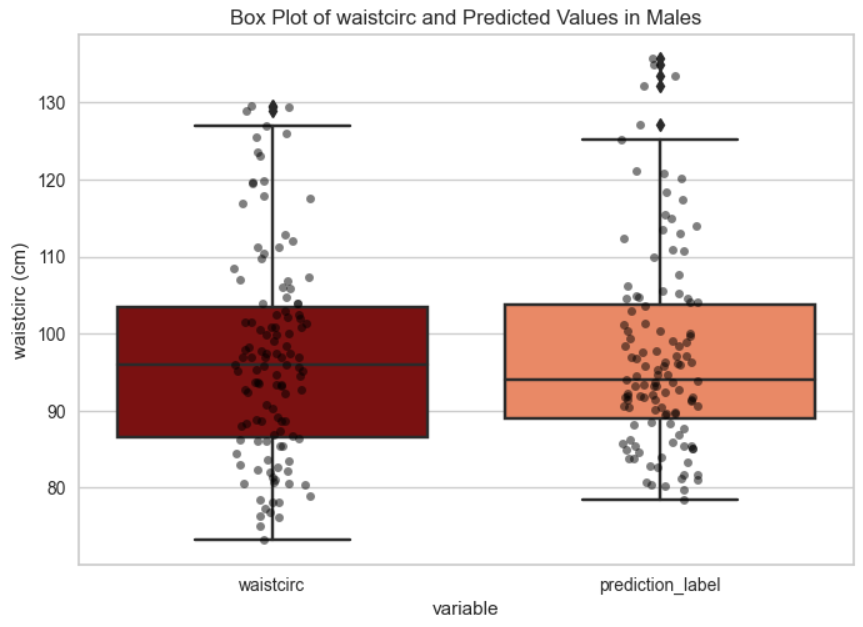


**Supplementary Table 3:** A summary table presenting the performance of the male model on both male and female data partitions, including corresponding p-values from t-tests.


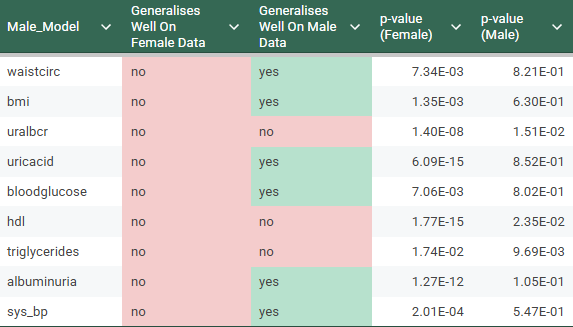


**Description:** H₀: The male-trained model predicted values without significant error. Hₐ: The male-trained model predicts values with significant error. H₀ is rejected if p < 0.05.
